# Surrogate endpoints for overall survival in chemotherapy and radiotherapy trials in operable and locally advanced lung cancer: a re-analysis of meta-analyses of individual patients' data

**DOI:** 10.1016/S1470-2045(13)70158-X

**Published:** 2013-06

**Authors:** Audrey Mauguen, Jean-Pierre Pignon, Sarah Burdett, Caroline Domerg, David Fisher, Rebecca Paulus, Samithra J Mandrekar, Chandra P Belani, Frances A Shepherd, Tim Eisen, Herbert Pang, Laurence Collette, William T Sause, Suzanne E Dahlberg, Jeffrey Crawford, Mary O'Brien, Steven E Schild, Mahesh Parmar, Jayne F Tierney, Cécile Le Pechoux, Stefan Michiels, on behalf of the Surrogate Lung Project Collaborative Group

**Affiliations:** a Meta-analysis Unit, Department of Biostatistics and Epidemiology, Gustave Roussy Institute, Villejuif, France; b Radiotherapy Department, Gustave Roussy Institute, Villejuif, France; c MRC Clinical Trials Unit, London, UK; d RTOG Statistical Center, Philadelphia, PA, USA; e Mayo Clinic and North Central Cancer Treatment Group, Rochester, MN, USA; f Penn State Hershey Cancer Institute, Hershey, PA, USA; g Princess Margaret Hospital and NCIC Clinical Trials Group, Toronto, ON, Canada; h Cambridge Biomedical Research Centre, Cambridge, UK; i Cancer and Leukemia Group B Statistical Center, Duke University, Durham, NC, USA; j EORTC Headquarters, Brussels, Belgium; k Intermountain Medical Center, Murray, UT, USA; l Dana-Farber Cancer Institute and Harvard School of Public Health, Boston, MA, USA; m Royal Marsden Hospital, London, UK; n Institut Jules Bordet, Université Libre de Bruxelles, Brussels, Belgium; o Université Paris-Sud 11, Paris France

## Abstract

**Background:**

The gold standard endpoint in clinical trials of chemotherapy and radiotherapy for lung cancer is overall survival. Although reliable and simple to measure, this endpoint takes years to observe. Surrogate endpoints that would enable earlier assessments of treatment effects would be useful. We assessed the correlations between potential surrogate endpoints and overall survival at individual and trial levels.

**Methods:**

We analysed individual patients' data from 15 071 patients involved in 60 randomised clinical trials that were assessed in six meta-analyses. Two meta-analyses were of adjuvant chemotherapy in non-small-cell lung cancer, three were of sequential or concurrent chemotherapy, and one was of modified radiotherapy in locally advanced lung cancer. We investigated disease-free survival (DFS) or progression-free survival (PFS), defined as the time from randomisation to local or distant relapse or death, and locoregional control, defined as the time to the first local event, as potential surrogate endpoints. At the individual level we calculated the squared correlations between distributions of these three endpoints and overall survival, and at the trial level we calculated the squared correlation between treatment effects for endpoints.

**Findings:**

In trials of adjuvant chemotherapy, correlations between DFS and overall survival were very good at the individual level (ρ^2^=0·83, 95% CI 0·83–0·83 in trials without radiotherapy, and 0·87, 0·87–0·87 in trials with radiotherapy) and excellent at trial level (*R*^2^=0·92, 95% CI 0·88–0·95 in trials without radiotherapy and 0·99, 0·98–1·00 in trials with radiotherapy). In studies of locally advanced disease, correlations between PFS and overall survival were very good at the individual level (ρ^2^ range 0·77–0·85, dependent on the regimen being assessed) and trial level (*R*^2^ range 0·89–0·97). In studies with data on locoregional control, individual-level correlations were good (ρ^2^=0·71, 95% CI 0·71–0·71 for concurrent chemotherapy and ρ^2^=0·61, 0·61–0·61 for modified *vs* standard radiotherapy) and trial-level correlations very good (*R*^2^=0·85, 95% CI 0·77–0·92 for concurrent chemotherapy and *R*^2^=0·95, 0·91–0·98 for modified *vs* standard radiotherapy).

**Interpretation:**

We found a high level of evidence that DFS is a valid surrogate endpoint for overall survival in studies of adjuvant chemotherapy involving patients with non-small-cell lung cancers, and PFS in those of chemotherapy and radiotherapy for patients with locally advanced lung cancers. Extrapolation to targeted agents, however, is not automatically warranted.

**Funding:**

Programme Hospitalier de Recherche Clinique, Ligue Nationale Contre le Cancer, British Medical Research Council, Sanofi-Aventis.

## Introduction

Worldwide, around 1·6 million new cases of lung cancer are diagnosed annually, which accounts for 13% of all diagnosed cancers and comprised, with 1·4 million deaths, 18% of cancer deaths in 2008. Lung cancer is the leading cause of cancer deaths in male patients.^1^ 80–85% of tumours are non-small-cell lung cancers (NSCLCs),^2^ which include adenocarcinomas, squamous-cell and large-cell carcinomas. The remaining 15–20% are small-cell lung cancers (SCLCs). Estimated 5-year survival in NSCLC is only 16%.^3^ Although surgery is generally viewed as the optimum treatment, only about 30% of patients qualify for potentially curative resection.^4^ A further 20%, mainly those presenting with locally advanced disease, undergo radical thoracic radiotherapy, with or without chemotherapy. The remaining 50% of patients, most of whom have late-stage or metastatic disease, generally receive palliative treatments.

The gold standard endpoint in randomised clinical trials of lung cancer is overall survival because it is simple to measure, easy to interpret, and measurement is unbiased. Some of the disadvantages of this endpoint are the need for long-term follow-up and large numbers of patients, the effect of successive treatment lines that might prolong survival, and the risk of non-cancer deaths rising with increasing time. Use of a surrogate endpoint at an earlier stage in clinical trials would speed up assessment of treatments and might reduce the cost of drug development. Between December, 1992, and July, 2010, the Food and Drug Administration granted accelerated approval for 35 oncology products on the basis of surrogate endpoints, such as disease-free survival ([DFS] eg, anastrozole in breast cancer) and progression-free survival ([PFS] eg, panitumumab in advanced colon cancer).^5^ In Europe in 2009, the European Medicines Agency approved gefinitib as first-line treatment in patients with locally advanced or metastatic EGFR-mutation-positive NSCLC, and extended the licence of erlotinib for this treatment in 2011, both on the basis of PFS.^6^, ^7^ Additionally, multiple second-line treatment options have become available, which encourages use of intermediate endpoints in studies of NSCLC.^8^

To be suitable, surrogate endpoints should predict overall survival, and the effect of treatments on the surrogate endpoints should predict their effects on overall survival in meta-analyses of individual patients' data.^9^ We have analysed trial data from five meta-analyses of individual patients' data by the NSCLC Meta-analyses Collaborative Group^10^, ^11^, ^12^, ^13^ and from one by the MAR-LC Collaborative Group^14^ to assess the use of DFS, PFS, and locoregional control as surrogate endpoints for overall survival.

## Methods

We assessed endpoints according to the following preplanned objectives: assess DFS as a surrogate endpoint for overall survival in patients with resectable NSCLC in trials studying the effect of adjuvant chemotherapy; assess PFS as a surrogate for overall survival in trials of chemotherapy and radiotherapy in patients with locally advanced lung cancer; and appraise time to locoregional control as a surrogate for overall survival in trials of radiotherapy or concurrent radiochemotherapy in locally advanced lung cancer. All analyses presented used the statistical methods described in the study protocol. All trials from the meta-analyses with available event data were included (appendix). All randomised patients were analysed according to intention to treat.

### Endpoint definitions

In all trials, data on events had been collected prospectively. Overall survival was defined as time from randomisation to death, irrespective of cause. Patients still alive at the last visit were censored as the date of last follow-up. DFS in patients suitable for surgery was defined as the time from randomisation to the first event (locoregional or distant recurrence or death from any cause). Patients without documented evidence of an event were censored at the date of last follow-up. PFS in patients unsuitable for surgery was defined as the time from randomisation to the first event (locoregional or distant progression or death). Patients who had no documented evidence of events were censored at the date of last follow-up. Locoregional control was defined as the time from randomisation to the first locoregional event. Patients with distant progression, who died or who had no documented evidence of distant progression or death were censored at the date of distant progression or last follow-up.

### Statistical analysis

Statistical analyses were done with SAS (version 9.2). The rank correlation coefficient ρ between distributions of the candidate surrogate endpoints and overall survival at the individual level was assessed with a bivariate survival model that takes censoring into account.^15^ The trial-level correlations between treatment effects (log hazard ratios) on the candidate surrogate endpoints and overall survival were quantified through a linear regression model,^16^ weighted by trial size. In trials of adjuvant chemotherapy, correlations between treatment effects on 3-year DFS and 5-year overall survival (with censoring of all patients at 3 years and 5 years) were assessed.^17^ In patients with locally advanced disease, correlations between 2-year PFS and 5-year overall survival were assessed.^18^ A sensitivity analysis assessed different cutoff points.

The squared correlation coefficients or coefficients of determination—ie, ρ^2^ at the individual level and *R*^2^ at the trial level—were calculated to investigate the amount of variation explained by the surrogate. The candidate surrogate endpoints were deemed acceptable only if both correlation coefficients were close to 1·00. We classified squared correlation values higher than 0·9 as excellent,^19^ higher than 0·75 as very good, higher than 0·5 as good, higher than 0·25 as moderate, and equal to or lower than 0·25 as poor.

The surrogate threshold effect was calculated, and was defined as the minimum treatment effect on the surrogate that would be necessary to predict a non-zero effect on overall survival.^20^ A future trial would require an upper limit of the CI for the estimated surrogate treatment effect to fall below the surrogate threshold effect to predict a non-zero effect on overall survival. The Kaplan-Meier method was used to calculate curves for DFS and overall survival.

For each meta-analysis, we used a leave-one-out cross-validation approach to assess the prediction accuracy of the surrogate model.^18^ Each trial was left out once and the linear model, weighted by trial size, was rebuilt with the other trials. This model was then applied to the left-out trial and a 95% prediction interval was calculated to compare the predicted and observed treatment effect on overall survival.

### Role of the funding source

The sponsors of the study had no role in study design, data collection, data analysis, data interpretation, or writing of the report. AM, J-PP, and SM had full access to all the raw data. The corresponding author had final responsibility for the decision to submit for publication.

## Results

This analysis is based on individual data for 15 071 patients in 60 randomised trials with long-term follow-up that were included in six meta-analyses (table 1).^11^, ^12^, ^13^, ^14^

**Table 1:** Description of trials by setting

	Number of patients (trials)*	**Disease-free or progression-free survival**		**Overall survival**	
		Number of events	Median (range) survival	Number of deaths	Median (range) survival
**Adjuvant therapy**					
Chemotherapy *vs* no chemotherapy	5379 (17)	2525	6·4 years (0–16·7)	2163	8·2 years (0–16·7)
Radiotherapy+chemotherapy *vs* radiotherapy alone	2247 (7)	1673	1·6 years (0–22·2)	1566	2·5 years (0–22·2)
**Locally advanced disease**					
Radiotherapy+sequential chemotherapy *vs* radiotherapy alone	1458 (8)	1375	7·9 months (0–213·6)	1333	11·7 months (0–213·6)
Radiotherapy+concurrent chemotherapy *vs* radiotherapy alone	2552 (15)	2391	8·1 months (0–189·6)	2305	14·1 months (0–189·6)
Radiotherapy+sequential chemotherapy *vs* radiotherapy+concurrent chemotherapy	1201 (6)	1094	8·3 months (0–134·4)	1065	14·6 months (0–134·4)
Modified radiotherapy *vs* standard radiotherapy	2685 (12)	2562	9·1 months (0–171·6)	2471	15·0 months (0–171·6)

* In multiarm trials, control patients and treatment comparisons are counted twice; the total number of unique patients is 15 071 and of unique trials is 60.

In the adjuvant setting, DFS data were available for 24 trials involving 7626 patients and investigating surgery compared with postoperative chemotherapy or no postoperative chemotherapy (median follow-up 5·7 years), or surgery plus postoperative chemotherapy and radiotherapy compared with surgery plus radiotherapy alone (median follow-up 6·4 years; table 1, appendix). Worse median DFS and overall survival in trials of postoperative radiotherapy and chemotherapy (table 1) reflected the poorer outlook of patients than in trials of chemotherapy alone.

In the locally advanced setting, PFS data were available for 29 chemotherapy trials involving 5211 patients. The studies assessed radiotherapy with or without concurrent (median follow-up 6·8 years) or sequential chemotherapy (median follow-up 5·2 years), or did a head-to-head comparison of radiotherapy plus sequential chemotherapy versus radiotherapy plus concurrent chemotherapy (median follow-up 6·1 years; table 1, appendix). Among these studies, locoregional control was assessed in 13 trials of radiotherapy with or without concurrent chemotherapy that involved 2123 patients. A further 12 trials were assessed in the locally advanced setting that compared standard radiotherapy with hyperfractionated or accelerated (modified) radiotherapy in 2685 patients (table 1, appendix). All 12 trials had data for PFS, and ten had data for locoregional control (2079 patients). Two of the trials enrolled only patients with SCLCs and the rest those with NSCLCs. The median follow-up time was 8·6 years. Cumulative percentages of events and additional descriptive statistics are shown in the appendix.

**Figure 1:**
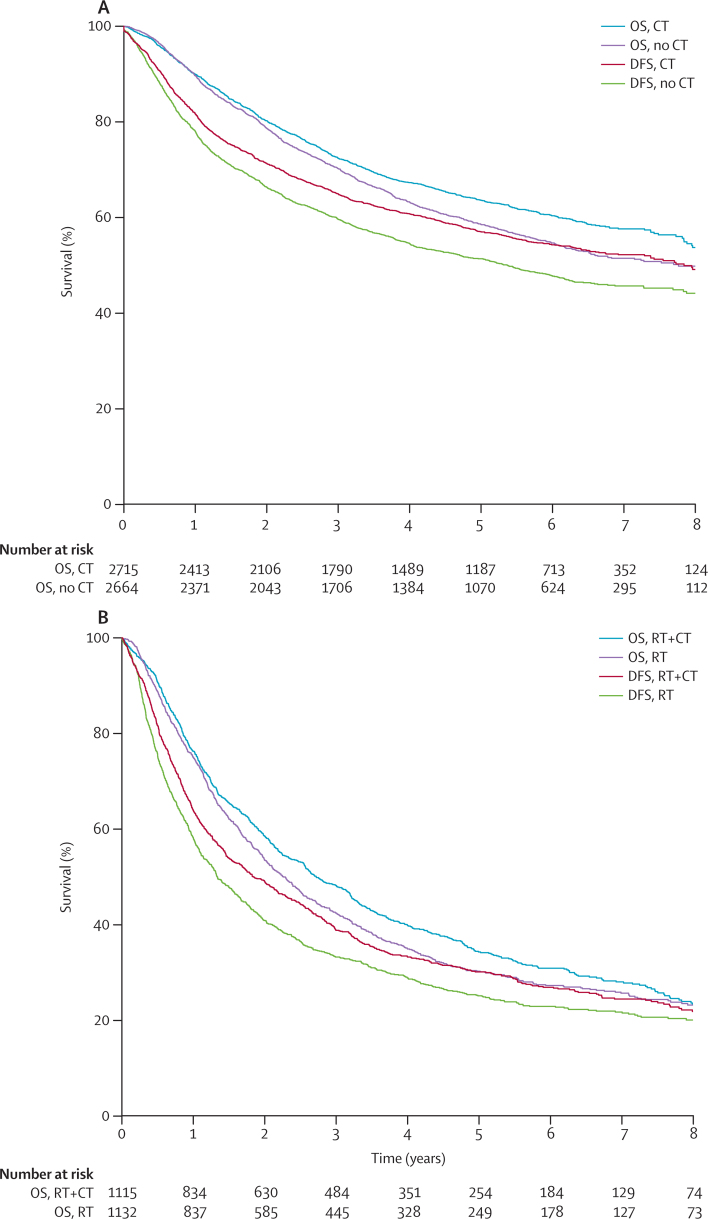
Kaplan-Meier curves of DFS and OS in assessment of adjuvant chemotherapy for non-small-cell lung cancers (A) Chemotherapy *vs* no chemotherapy. (B) Radiotherapy+chemotherapy *vs* radiotherapy alone. OS=overall survival. CT=chemotherapy. DFS=disease-free survival. RT=radiotherapy.

**Figure 2:**
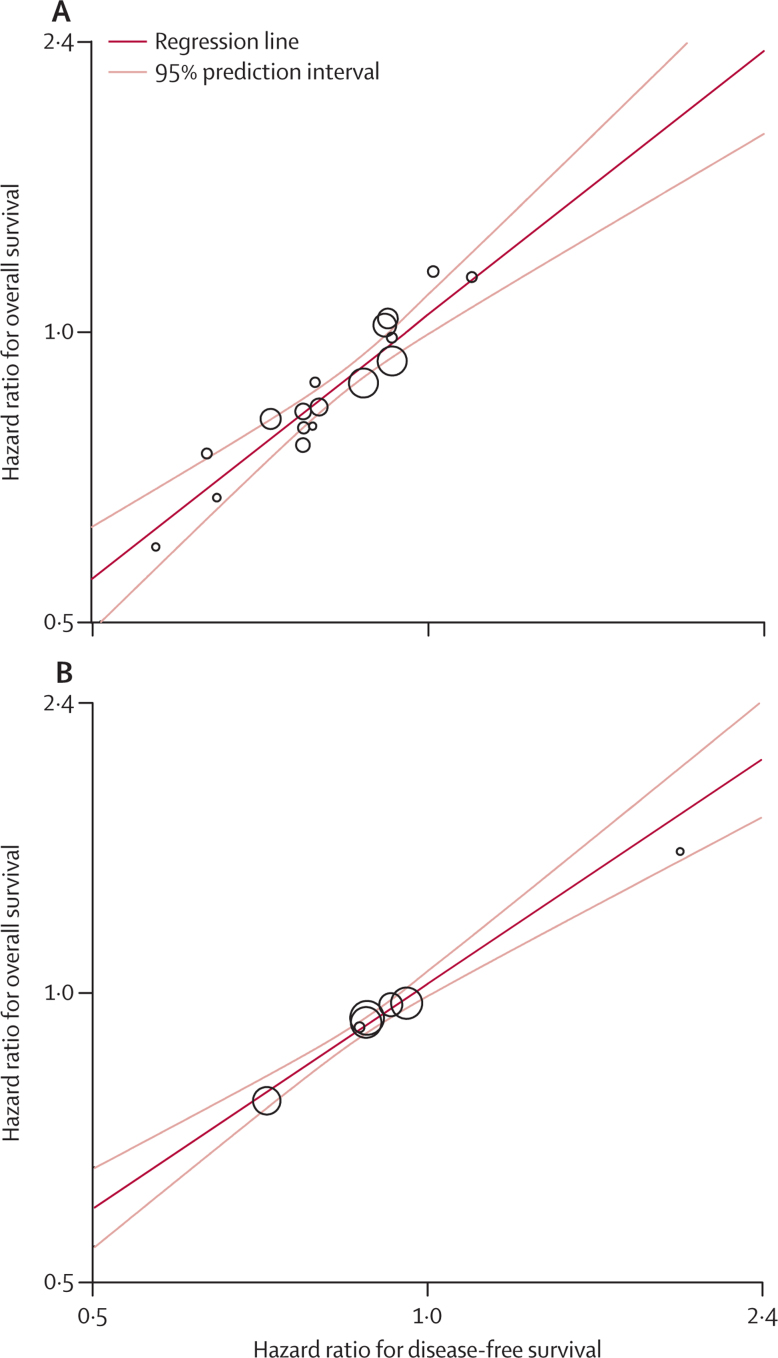
Correlation between treatment effects on disease-free and overall survival in the assessment of adjuvant treatment for non-small-cell lung cancers (A) Chemotherapy compared with no chemotherapy. (B) Radiotherapy plus chemotherapy compared with radiotherapy alone. Each trial is represented by a circle, with a size proportional to the number of patients. A logarithmic scale is used on axes. Correlation values are excellent (*R*^2^=0·92 and *R*^2^=0·99).

A strong association was noted between DFS and overall survival in patients with NSCLC who received adjuvant chemotherapy with or without radiotherapy (ρ^2^=0·83, 95% CI 0·83–0·83 for chemotherapy *vs* no chemotherapy, and 0·87, 0·87–0·87 for those comparing radiotherapy plus chemotherapy *vs* radiotherapy alone; figure 1, table 2). The squared correlations between the treatment effects on DFS and those on overall survival were excellent (*R*^2^=0·92, 95% CI 0·88–0·95 and 0·99, 0·98–1·00; table 2). Linear regression showed excellent correlation between the treatment effects on DFS and those on overall survival (figure 2, appendix). The sensitivity analysis, which aimed to reflect typical trial conditions and to correlate the treatment effects estimated on DFS at 3 years with those on overall survival at 5 years, yielded slightly lower *R*^2^ values, but these were still very good to excellent (0·88, 0·83–0·93 for chemotherapy *vs* no chemotherapy and 0·96, 0·93–0·99 for radiotherapy plus chemotherapy *vs* radiotherapy alone; table 2). 79% and 84% of DFS events, respectively, occurred in the first 3 years (appendix). An exploratory analysis with earlier cutoff times for DFS suggested that after only 2 years, treatment effects on DFS already correlated well with those on overall survival at 5 years (appendix). Surrogate threshold effects for DFS were 0·88 for chemotherapy compared with no chemotherapy and 0·95 for radiotherapy plus chemotherapy compared with radiotherapy alone.

**Table 2:** Individual and trial-level correlation coefficients and sensitivity data for DFS and PFS

	**Number of patients (trials)**	**DFS or PFS *vs* overall survival**		**3-year DFS or 2-year PFS *vs* 5-year overall survival (*R*^2^ [95% CI])**
		Individual level (ρ^2^ [95% CI])	Trial level (*R*^2^ [95% CI])	
**Adjuvant therapy**				
Chemotherapy *vs* no chemotherapy	5379 (17)	0·83 (0·83–0·83)	0·92 (0·88–0·95)	0·88 (0·83–0·93)
Radiotherapy+chemotherapy *vs* radiotherapy alone	2247 (7)	0·87 (0·87–0·87)	0·99 (0·98–1·00)	0·96 (0·93–0·99)
**Locally advanced disease**				
Radiotherapy+sequential chemotherapy *vs* radiotherapy alone	1458 (8)	0·77 (0·77–0·77)	0·96 (0·93–0·99)	0·77 (0·63–0·91)
Radiotherapy+concurrent chemotherapy *vs* radiotherapy alone	2552 (15)	0·85 (0·85–0·85)	0·97 (0·96–0·99)	0·95 (0·92–0·97)
Radiotherapy+sequential chemotherapy *vs* radiotherapy+concurrent chemotherapy	1201 (6)	0·83 (0·83–0·83)	0·89 (0·81–0·97)	0·75 (0·58–0·92)
Modified radiotherapy *vs* standard radiotherapy	2685 (12)	0·81 (0·81–0·81)	0·96 (0·93–0·98)	0·85 (0·78–0·93)

DFS=disease-free survival. PFS=progression-free survival.

**Figure 3:**
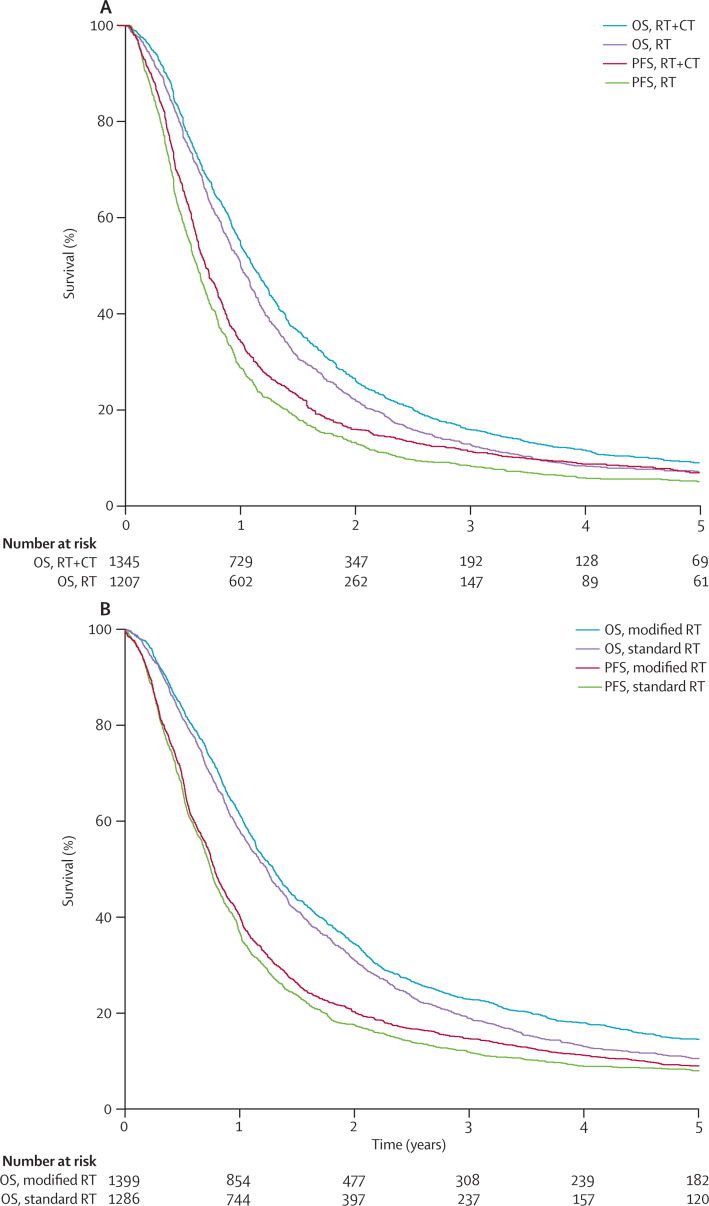
Kaplan-Meier curves of PFS and OS in the treatment of locally advanced disease (A) Radiotherapy plus concurrent chemoradiotherapy compared with radiotherapy alone in non-small-cell lung cancer. (B) Modified radiotherapy compared with standard radiotherapy in non-small-cell and small-cell lung cancers. OS=overall survival. RT=radiotherapy. CT=chemotherapy. PFS=progression-free survival.

**Figure 4:**
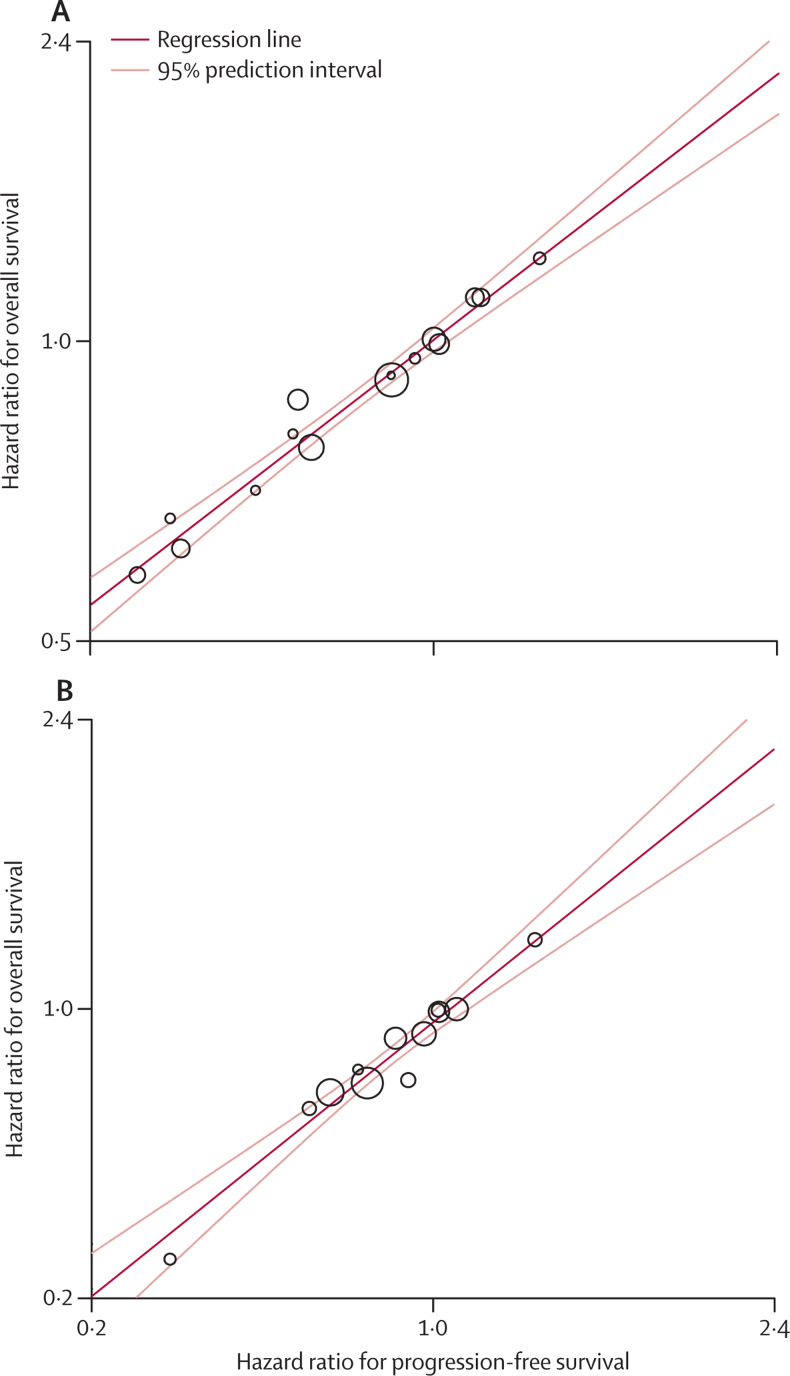
Correlation between treatment effects on progression-free and overall survival in locally advanced disease (A) Radiotherapy plus concurrent chemotherapy compared with radiotherapy alone in non-small-cell lung cancer. (B) Modified radiotherapy compared with standard radiotherapy in non-small-cell and small-cell lung cancers. Correlation values are excellent (*R*^2^=0·97 and *R*^2^=0·96).

Survival curves for PFS and overall survival in locally advanced disease for radiotherapy plus concurrent chemotherapy compared with radiotherapy alone, and for modified radiotherapy compared with standard radiotherapy are shown in figure 3. Very good correlation values were noted for locally advanced disease (ρ^2^=0·77–0·85, table 2). Treatment effects on PFS correlated well from very good to excellent with those on overall survival (*R*^2^=0·89–0·97, table 2). Linear regression confirmed the strength of the correlation (figure 4). In the sensitivity analysis, PFS at 2 years correlated reasonably strongly at the trial level with overall survival (very good to excellent; table 2, appendix). The surrogate threshold effects for PFS were 0·93 for radiotherapy plus sequential chemotherapy compared with radiotherapy alone, 0·95 for radiotherapy plus concurrent chemotherapy compared with radiotherapy alone, 0·90 for radiotherapy plus sequential chemotherapy compared with radiotherapy plus concurrent chemotherapy, and 1·00 for modified radiotherapy compared with standard radiotherapy.

With respect to locoregional control in the trials of radiotherapy plus concurrent chemotherapy compared with radiotherapy alone and of modified radiotherapy compared with standard radiotherapy, squared individual-level correlations with overall survival were good (ρ^2^ 0·71, 95% CI 0·71–0·71 and 0·61, 0·61–0·61) and the squared treatment-effect correlations were very good (*R*^2^ 0·85, 95% CI 0·77–0·92 and 0·95, 0·91–0·98; appendix).

**Figure 5:**
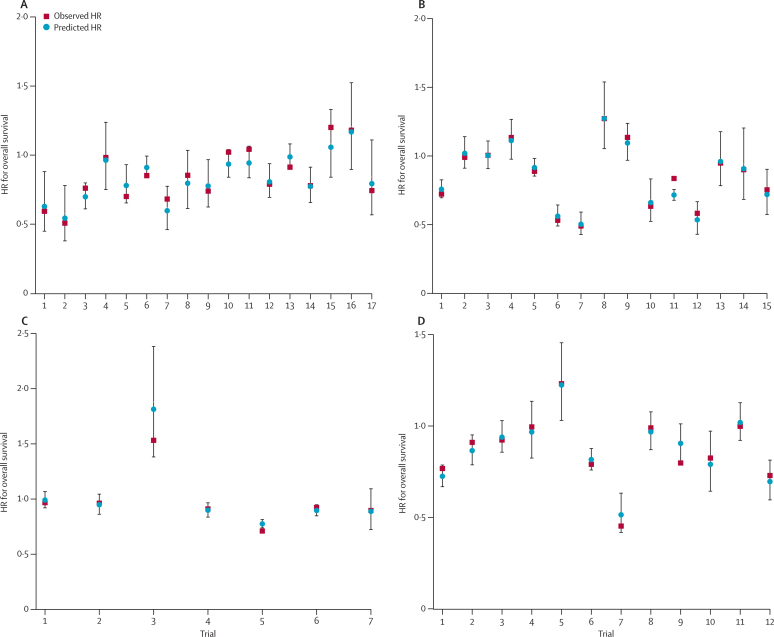
Internal validation of the prediction of overall survival by treatment effects on surrogate endpoints (A) Treatment effects on disease-free survival for adjuvant chemotherapy compared with no chemotherapy in non-small-cell lung cancer. (B) Treatment effects on progression-free survival effects for radiotherapy plus concurrent chemotherapy compared with radiotherapy alone in non-small-cell lung cancer. (C) Treatment effects on disease-free survival for radiotherapy plus adjuvant chemotherapy compared with radiotherapy alone in non-small-cell lung cancer. (D) Treatment effects on progression-free survival for modified radiotherapy compared with standard radiotherapy in non-small-cell and small-cell lung cancers. Predicted HRs for overall survival are calculated from the observed HR on disease-free or progression-free survival of that particular trial and the surrogate model built on all the other trials. Observed HRs are shown for overall survival. All values are shown with 95% prediction intervals. HR=hazard ratio.

The prediction results from the cross-validation analyses showed that for DFS in the adjuvant setting, the hazard ratios for overall survival fell within the 95% prediction intervals in all 17 trials for chemotherapy compared with no chemotherapy and in six of seven trials of radiotherapy plus chemotherapy compared with radiotherapy alone (figure 5). For PFS in the locally advanced setting, the observed hazard ratio for overall survival fell between the limits of the 95% prediction intervals in all eight trials for radiotherapy plus sequential chemotherapy compared with radiotherapy alone, 14 of 15 trials of radiotherapy plus concurrent chemotherapy compared with radiotherapy alone, all six trials for radiotherapy plus sequential chemotherapy compared with radiotherapy plus concurrent chemotherapy, and in 11 of 12 trials of modified radiotherapy compared with standard radiotherapy (figure 5, appendix).

## Discussion

Our assessment of a large sample of data for patients with lung cancer provides high-level evidence that DFS is a valid surrogate endpoint for overall survival in studies of adjuvant chemotherapy in patients with NSCLC, and that PFS is a valid surrogate in assessment of chemotherapy and radiotherapy in patients with locally advanced disease. DFS and PFS should, therefore, be considered for use as primary endpoints for chemotherapy and radiotherapy trials (panel).

Panel **Research in context**

**Systematic review**

The gold standard endpoint in randomised clinical trials of lung cancer is overall survival because it is simple to measure, easy to interpret, and measurement is unbiased. Some of the disadvantages of this endpoint are the need for long-term follow-up and large numbers of patients, the effect of successive treatment lines that might prolong survival, and the risk of non-cancer deaths rising with increasing time. Use of a surrogate endpoint at an earlier stage in clinical trials would speed up assessment of treatments and might reduce the cost of drug development. We have analysed trial data from five meta-analyses of individual patients' data by the NSCLC Meta-analyses Collaborative Group^10^, ^11^, ^12^, ^13^ and from one by the MAR-LC Collaborative Group^14^ to assess the use of disease-free survival, progression-free survival, and locoregional control as surrogate endpoints for overall survival.

**Interpretation**

Disease-free survival may be used as a primary endpoint in adjuvant chemotherapy trials involving patients with non-small-cell lung cancers, and progression-free survival is suitable for use in trials of chemotherapy and radiotherapy in patients with locally advanced lung cancer. When progression-free survival is substituted for overall survival, a trade-off is found between earlier results and possible biases in the assessment of progressions. These results do not automatically translate to targeted agents.

Overall, the correlations for DFS or PFS indicated that almost 89–99% of the variation in treatment effects on overall survival can be explained by effects on DFS or PFS. The cross-validation results confirmed the accurate prediction of the treatment effect on overall survival based on the effects observed on DFS or PFS. Correlations for locoregional control were good at the individual level and very good at the trial level, but were a little lower than the corresponding values for PFS. Since the number of events with locoregional control is always lower than for PFS in randomised clinical trials for the same sample size and follow-up, the statistical power for the assessment of locoregional control will also always be lower.

The sensitivity analysis strongly suggests that the information on DFS and PFS acquired at 3 years and 2 years, respectively, could be sufficient to predict the 5-year effect of treatment on overall survival in patients with operable and locally advanced tumours. We recommend the use of DFS and PFS as time-to-event outcomes in randomised clinical trials rather than measurement at a specific timepoint.

We used DFS or PFS as defined by the investigators in the trials, which included patients across a wide time range, but the consistency of the results across the meta-analyses is reassuring. The potential gain in the use of PFS as a surrogate in locally advanced lung cancer is probably smaller than that associated with the use of DFS to assess adjuvant treatment because the times from progression to death are shorter. The possible gains of using PFS should, therefore, be weighed against the possible risks and the known biases associated with assessing progression^21^ and the difficulties in assessing local relapses after chemotherapy or chemoradiation.

Use of a similar approach to assess individual patients' data has shown that DFS is useful as a surrogate for overall survival in the testing of adjuvant chemotherapy regimens for other cancer types, such as colorectal cancer, trial-level *R*^2^=0·85 (very good).^17^ The data for PFS, however, have been less consistent, although excellent correlation (*R*^2^=0·98) was seen with overall survival in assessment of fluoropyrimidine-based chemotherapy in advanced colorectal cancer.^22^ A combined DFS and PFS endpoint proved to be an appropriate surrogate for the assessment of various chemotherapy (*R*^2^=0·87) and radiotherapy (*R*^2^=0·96) regimens in locally advanced head and neck cancers.^18^ PFS alone, however, was not a valid surrogate for overall survival in trials comparing an anthracycline with a taxane for advanced breast cancer (*R*^2^=0·23).^23^ Few surrogacy assessments based on individual patient data have been done in lung cancer. In advanced NSCLC, treatment effects on PFS were moderately correlated with those on overall survival in trials comparing first-line docetaxel with vinorelbine (*R*^2^=0·38).^24^ In three trials of patients with extensive SCLC, PFS was strongly associated with overall survival at the trial level (*R*^2^=0·79).^25^ Part of the discrepancy between the NSCLC and SCLC results could be due to the administration of subsequent lines of therapy that prolong survival in patients with NSCLCs.

Interpretation of outcomes on the basis of surrogate endpoints has several limitations. A surrogate endpoint can only be validated for the therapies assessed; extrapolation to treatments administered by different methods or with substantially different mechanisms of action might not be warranted. For instance, for targeted agents, surrogate endpoints will need to be studied directly in trials of the agents. Additionally, we reassessed data from a generation of trials in which chemotherapy or radiotherapy were the main treatments being investigated and when few, if any, active agents were available as subsequent lines of treatment. The use of subsequent treatments can be an important confounding factor to the effectiveness of surrogate endpoints. Also, crossover can affect the power of assessing effects on overall survival, particularly when an effective second-line treatment is available.^26^, ^27^

The use of DFS or PFS as primary endpoints in future clinical trials does not reduce the need for long-term follow-up of patients. In many cases it remains necessary to control for unexpected adverse reactions and to assess overall survival, even when crossover to the experimental therapy from the control arm is permitted. We stress that overall survival remains the most valid available endpoint. The use of validated surrogates, however, would enable earlier assessments of treatment effects and could lead to conditional approval by regulatory authorities without premature discontinuation of follow-up, which is particularly important to assess the potential for long-term late or toxic effects when a proportion of patients are cured.^28^
