# Supplementary material for: Surrogate endpoints for overall survival in chemotherapy and radiotherapy trials in operable and locally advanced lung cancer: a re-analysis of meta-analyses of individual patients' data
Source: Lancet Oncol. 2013 Jun;14(7):619–26. doi: 10.1016/S1470-2045(13)70158-X (PMC3732017; doi:10.1016/S1470-2045(13)70158-X)
Supplement: Supplementary appendix [file mmc1.pdf]

THE LANCET Oncology

Supplementary appendix

This appendix formed part of the original submission and has been peer reviewed. We post it as supplied by the authors.

Supplement to: Mauguen A, Pignon J-P, Burdett S, et al. Surrogate endpoints for overall survival in chemotherapy and radiotherapy trials in operable and locally advanced lung cancer: a re-analysis of meta-analyses of individual patients' data. *Lancet Oncol* 2013; published online May 14. http://dx.doi.org/10.1016/S1470-2045(13)70158-X.

List of contributors:

Surrogate Lung Project Collaborative Group Secretariat

S. Burdett, D. Fisher, C. Le Péchoux, A. Mauguen, S. Michiels, J.P. Pignon, J.F. Tierney.

Surrogate Lung Project Collaborative Group Advisory Board

C.P.Belani, L. Collette, S. Dahlberg, T. Eisen, S.Mandrekar, M. O'Brien, M. Parmar, H. Pang, R. Paulus, J. Crawford, W. Sause, S.E. Schild, F. Shepherd.

Collaborators (listed alphabetically)

R. Arriagada (Institut Gustave Roussy, Villejuif, France; Karolinska Institutet, Stockholm, Sweden),

S. Atagi (Kinki-Chuo Chest Medical Center, Japan),

A. Auperin (InstitutGustave-Roussy, Villejuif, France),

D. Ball (Peter MacCallum Cancer Centre, East Melbourne, Australia),

M. Baumann (University Hospital/Medical Faculty, Carl Gustav Carus Technische Universität Dresden and Helmholtz Center Dresden-Rossendorf, Dresden, Germany),

K. Behrendt (Maria Sklodowska–Curie Memorial Cancer Center, Gliwice Branch, Poland),

J. Belderbos (Netherlands Cancer Institute, Amsterdam, the Netherlands),

B. Bergman (Sahlgrenska Academy, Gothenburg, Sweden),

J.F. Bishop (VictorianComprehensive Cancer Centre, Melbourne, Australia),

J.A. Bonner (University of Alabama-Birmingham, AL, USA),

M.C. Bozonnat (Université Montpellier I, Montpellier, France),

H. Choy (The University of Texas Southwestern, Dallas, Tx, USA)

G. Clamon (University of Iowa Hospital and Clinics, Iowa City, IO, USA),

W.J. Curran (Winship Cancer Institute of Emory University, Atlanta, USA),

D. De Ruysscher (University Hospitals Leuven/ KU Leuven, Belgium),

B. Dautzenberg (Groupe Hospitalier Pitié Salpêtrière, Paris),

R.O. Dillman (Hoag Cancer Center, Newport Beach, CA, USA),

S. Dische (Mount Vernon Hospital, Northwood Middlesex, UK),

J.Y. Douillard (Centre Rene Gauducheau, Nantes, France),

A. Dunant (Institut Gustave-Roussy, Villejuif, France),

P. Fournel (Institut de Cancérologie Lucien Neuwirth, Saint Etienne, France),

K. Furuse (Japan-Multinational Trial Organization, Kyoto, Japan),

S. Geyer (Mayo Clinic and NCCTG, Rochester, MN, USA),

H.J.M. Groen (University Hospital Groningen, The Netherlands),

P.A. Helle (Medisch Spectrum Twente, Enschede, The Netherlands),

M. Henry-Amar (Centre François Baclesse, Caen, Françe),

J.E. Herndon (Duke University Medical Center, Durham, NC, USA),

J.P. Higgins (MRC Biostatistics Unit, Cambridge, UK),

R.M. Huber (University of Munich & Thoracic Oncology Centre Munich, Germany),

M. Imaizumi (Nagoya University School of Medicine, Nagoya, Japan),

B. Jeremic (The University Hospital, Kragujevac, Serbia),

D.H. Johnson (Vanderbilt-Ingram Cancer Center, Nashville, USA),

H. Kato (Tokyo Medical University, Tokyo, Japan),

S.M. Keller (Montefiore Medical Center, NY, USA),

A. Knuuttila (Helsinki University Central Hospital, Helsinki, Finland),

R. Koch (University Hospital and Medical Faculty Carl Gustav Carus, Dresden, Germany),

CCE. Koning (The Academic Medical Center, Amsterdam, The Netherlands),

C. Legrand (Université Catholique de Louvain, Louvain-La-Neuve, Belgium),

T. Le Chevalier (InstitutGustave-Roussy, Villejuif, France),

K. Mattson (Helsinki University Central Hospital, Helsinki, Finland),

T. Mineo (Policlinico Tor Vergata University, Rome, Italy),

F. Mornex (Centre Hospitalier Lyon Sud, Pierre Bénite, France),

M. Nankivell (MRC Clinical Trials Unit, London, UK),

J.H. Park (Korea Cancer Center Hospital, Seoul, South Korea),

R. Paulus (RTOG Statistical Center Philadelphia, PA, USA),

E. Rolland (InstitutGustave-Roussy, Villejuif, France),

R. Rosell (Catalan Institute of Oncology, Hospital Germans Trias i Pujol, Barcelona, Spain),

S. Sarihan (Uludag University, Turkey),

M.I. Saunders ((Mount Vernon Hospital, Northwood Middlesex, UK),

G.V. Scagliotti (S. Luigi Hospital, Torino, Italy),

M. Schaapveld (Comprehensive Cancer Center North, The Netherlands),

L. Seymour (Queen's University, NCIC Clinical Trials Group, Kingston, ON, Canada), F.A. Shepherd (Princess Margaret Hospital, Toronto, ON, Canada),

T. Shibata (JCOG Data Center, National Cancer center, Tokyo, Japan),

R.L. Souhami (Cancer Research UK, London, UK),

S.G. Spiro (University College Hospital, London, UK),

R.J. Stephens (MRC Clinical Trials Unit, London, UK),

L.A. Stewart (University of York, York, UK),

G.M. Strauss (Tufts Medical Center, Boston, MA, USA),

R. Sylvester (EORTC Headquarters, Brussels, Belgium),

H. Tada (Osaka City General Hospital, Osaka, Japan),

F. Tanaka (Hyogo College of Medicine, Nishinomiya),

V. Torri (Istituto di RicercheFarmacologiche "Mario Negri", Milan, Italy),

A.T. Turrisi (Sinai Grace Hospital, Detroit, MI, USA),

A. Uitterhoeve (Academic Medical Center, Amsterdam, the Netherlands),

K. Ulm (InstitutfürMedizinischeStatistik und Epidemiologie, Munich, Germany),

H.C. Ulutin (GATA Radiation Oncology Department, Ankara, Turkey),

J. van Meerbeeck (Ghent University & Antwerp University Hospital, Belgium)

H. Wada (Kyoto University, Kyoto, Japan),

D. Waller (Glenfield Hospital, Groby Road, Leicester),

X.F. Wang (Duke University Medical Center, Durham, NC, USA),

K. Winter (RTOG Statistical Center Philadelphia, PA, USA),

G.C. Xu (Sun Yat-Sen University Cancer Center, Guangzhou, China),

T. Yamanaka (National Kyushu Cancer Center, Fukuoka, Japan),

A. Zajusz (Maria Sklodowska–Curie Memorial Cancer Center, Gliwice Branch, Poland).

Contents of appendix:

Appendix A1: Description of trials – operable patients 4

Appendix A2: Description of trials – locally advanced patients, sequential CT 7

Appendix A3: Description of trials – locally advanced patients, concurrent CT 8

Appendix A4: Description of trials – locally advanced patients, sequential versus concurrent CT 10

Appendix A5: Description of trials – radiotherapy trials 11

Appendix A6: Cumulative percentage of DFS/PFS events 13

Appendix A7: Summary of data 15

Appendix A8: Surrogacy analyses results 17

Appendix A9: Sensitivity analysis: different cut-off points 26

Appendix A10: Leave-one out crossvalidation results 27

Appendix A1: Description of trials – operable patients

Data on disease-free survival was available for 64% of the patients in the trials comparing CT vs. no CT and 84 % of the patients in the trials comparing RT + CT vs. RT (CT: chemotherapy, RT: radiotherapy).

Trial	Period of recruitment	Number of patients (in surrogate analysis)	Drugs used and dose per cycle (mg/m²)	Radiotherapy dose (Gy) / fractions
**Operable NSCLC without post-operative RT**				
WJSG 3[A1-1]	1988-89	225 (224)	Cisplatin 80 + Vindesine (2–3; once or twice), Mitomycin 8;<br>2 cycles<br>Tegafur + Uracil (400 total)<br>Daily treatment for 1 year	None
Mineo [A1-2]	1988-94	66 (66)	Cisplatin 100 + Etoposide 120<br>6 cycles	None
Xu [A1-3]	1989-92	70 (70)	Cisplatin 100 + Cyclophosphamide 300 + Vincristine 1·4 + Doxorubicin 50 + Lomustine 50<br>4 cycles<br>oral Tegafur (600–900 total)<br>Daily treatment for 1 year	None
Park1 [A1-4]	1989-98	118 (118)	Cisplatin 100 + Mitomycin C 10 + Vinblastine 6<br>3-4 cycles	None
Park2 [A1-5]	1989-98	108 (107)	Cisplatin 100 + Mitomycin C 10 + Vinblastine 6<br>3-4 cycles	None
ACTLC4A [A1-6]	1992-95	208 (155)	Cisplatin 80<br>1 cycles<br>Vindesine 3 (twice)<br>2 cycles<br>Tegafur + Uracil 400<br>Daily treatment for 2 years	None
JLCRG [A1-7]	1994-97	999 (999)	Tegafur + Uracil 250 mg/m2<br>Daily treatment for 2 years	None
ALPI1 [A1-8]	1994-99	618 (618)	Cisplatin 100 + Vindesine 3 + Mitomycin C 8<br>3 cycles	None
JCOG 9304 [A1-9]	1994-99	119 (119)	Cisplatin 80 + Vindesine 3<br>3 cycles	None
ANITA1[A1-10]	1994-00	463 (463)	Cisplatin 100 + Vinorelbine 30<br>4 cycles	None
JBR10 [A1-11]	1994-01	482 (482)	Cisplatin 50 + Vinorelbine 25<br>4 cycles	None
IALT1 [A1-12]	1995-01	1001 (1001)	Cisplatin (80, 100, or 120) + Vindesine (3; weekly then twice weekly);<br>3-4 cycles	None
IALT2 [A1-12]	1995-01	294 (294)	Cisplatin (80, 100, or 120), + Vinorelbine (30; weekly)<br>3-4 cycles	None
BLT1 [A1-13]	1995-01	136 (136)	Cisplatin 50 + Mitomycin 6 + vinblastine 6<br>or<br>Cisplatin 80 + Vindesine 6<br>3 cycles	None

BLT2 [A1-13]	1995-01	65 (65)	Cisplatin 50 + Mitomycin 6 + Ifosphamide 3<br>3 cycles	None
BLT3 [A1-13]	1995-01	118 (118)	Cisplatin 50 + Mitomycin C 6 + Ifosphamide 3<br>3 cycles	None
CALGB 9633 [A1-14]	1996–2003	344 (344)	Carboplatin (6 mg/mL over 45–60 min) + Paclitaxel 200<br>4 cycles	None
**Operable NSCLC with post-operative RT**				
GETCB 01CB82 [A1-15]	1982-86	267 (267)	Cisplatin 75 + Doxorubicin 40 + Vincristine 1.2 +<br>Lomustine 80* alternating with Cyclophosphamide 600<br>3 cycles	60-65 / 30-33<br>After chemotherapy
EORTC 08861[unpublished]	1986-90	24 (24)	Cisplatin 100 + Vindesine 6<br>4 cycles	56 / 28<br>After 2 cycles<br>Concurrent for 2 cycles
Int 0115[A1-16]	1991-97	488 (488)	Cisplatin 60 + Etoposide 120*3<br>4 cycles	50.4 / 28<br>Concurrent
ALPI3 [A1-8]	1994-99	470 (470)	Cisplatin 100 + Vindesine 3*2 + Mitomycin 8<br>3 cycles	50-54 / 25-27<br>After chemotherapy
ANITA3 [A1-10]	1994-00	377 (377)	Cisplatin 100 + Vinorelbine 30*4<br>4 cycles	45-60 / 23-30<br>After chemotherapy
BLT4[A1-13]	1995-01	49 (49)	MIC: Cisplatin 50, Mitomycin 6, Ifosfamide 3<br>MVP: Cisplatin 50, Mitomycin 6, Vinblastine 6<br>CV: Cisplatin 80, Vindesine 3*2<br>NP: Cisplatin 80, Vinorelbine 30*2<br>3 cycles	40-60 / 15-30<br>After chemotherapy
IALT3-4 [A1-12]	1995-01	572 (572)	Cisplatin 80, 100 or 120 + Vindesine 3*2 N=16<br>or Vinblastine 4*2 N=119<br>or Vinorelbine 30 weekly N=206<br>or Etoposid 30*3 N=231<br>3 or 4 cycles	≤ 60<br>After chemotherapy

* Total dose

ACTLC: Study Group of Adjuvant Chemotherapy for Lung Cancer. ALPI: Adjuvant Lung Cancer Project Italy. ANITA: Adjuvant Navelbine International Trialist Association. BLT: Big Lung Trial. CALGB: Cancer and Leukemia Group B. EORTC: European Organization for Research and Treatment of Cancer. GETCB : Groupe d'Etude et de Traitement des Cancers Bronchiques. IALT: International Adjuvant Lung Trial. Int = US intergroup trial. JBR10: National Cancer Institute of Canada Clinical Trial Group trial JBR10. JCOG: Japan Clinical Oncology Group. JLCRG: Japan Lung Cancer Research Group. WJSG: West Japan Study Group for Lung Cancer Surgery.

Gy: Grays; NSCLC: non-small cell lung cancer; RT: radiotherapy

1. Wada H et al. West Japan Study Group for lung cancer surgery. Post-operative adjuvant chemotherapy with PVM (cisplatin + vindesine + mitomycin c) and UFT (uracil and tegaful) in resected stage I-II NSCLC (non-small cell lung cancer): a randomised clinical trial. Eur J Cardiothorac Surg 1999; 15: 438–43.
2. Mineo TC, Ambrogi V, Corsaro V, Roselli M. Postoperative adjuvant therapy for stage IB non-small cell lung cancer. Eur J Cardiothoracic Surg 2001; 20: 378–84.
3. Xu G, Rong T, Lin P. Adjuvant chemotherapy following radical surgery for non-small cell lung cancer: a randomized study. Zhonghua Zhong Liu Za Zhi 1998; 20: 228–30.
4. Park JH, Lee C-T, Lee HW, Baek HJ, Zo JI, Shim YM. Postoperative adjuvant chemotherapy for stage I non-small cell lung cancer. Eur J Cardiothorac Surg 2005; 27: 1086–91.
5. Park JH. Postoperative adjuvant therapy for stage IIIA non-small cell lung cancer. J Thorac Oncol 2007; 2 (suppl): S651.
6. Imaizumi M. Postoperative adjuvant cisplatin, vindesine, plus uracil-tegafur chemotherapy increased survival of patients with completely resected p-stage I non-small cell lung cancer. Lung Cancer 2005; 49: 85–94.
7. Kato H, Ichinose Y, Ohta M, et al. A randomised trial of adjuvant chemotherapy with uracil-tegafur for adenocarcinoma of the lung. N Engl J Med 2004; 350: 1713–21.

8. Scagliotti GV, Fossati R, Torri V, et al. Randomized study of adjuvant chemotherapy for completely resected stage I, II or IIIa non-small cell lung cancer. J Natl Cancer Inst 2003; 95: 1453–61.

9. Tada H, Tsuchiya R, Ichinose Y, et al. A randomized trial comparing adjuvant chemotherapy versus surgery alone for completely resected pN2 non-small cell lung cancer (JCOG 9304). Lung Cancer 2004; 43: 167–73.

10. Douillard JY, Rosell R, De Lena M, et al. Adjuvant vinorelbine plus cisplatin versus observation in patients with completely resected stage IB–IIIA non-small-cell lung cancer (Adjuvant Navelbine International Trialist Association [ANITA]): a randomised controlled trial. Lancet Oncol 2006; 7: 719–27.

11. Winton T, Livingston R, Johnson D, et al. Vinorelbine plus cisplatin vs observation in resected non-small cell lung cancer. N Engl J Med 2005; 352: 2589–97.

12. The International Adjuvant Lung Cancer Trial Collaborative Group. Cisplatin-based adjuvant chemotherapy in patients with completely resected non-small cell lung cancer. N Engl J Med 2004; 350: 351–60.

13. Waller D, Peake MD, Stephens RJ, et al. Chemotherapy for patients with non-small cell lung cancer: the surgical setting of the Big Lung Trial. Eur J Cardiothorac Surg 2004; 26: 173–82.

14. Strauss GM, Herndon JE 2nd, Maddaus MA, et al. Adjuvant paclitaxel plus carboplatin compared with observation in stage IB non-small-cell lung cancer: CALGB 9633 with the Cancer and Leukemia Group B, Radiation Therapy Oncology Group, and North Central Cancer Treatment Group Study Groups. J Clin Oncol 2008; 26: 5043–51.

15. Dautzenberg B, Chastang C, Arriagada R, Le Chevalier T, Belpomme D, Hurdebourcq M et al. Adjuvant radiotherapy versus combined sequential chemotherapy followed by radiotherapy in the treatment of resected non-small cell lung cancer. Cancer 1995; 76:779-786.

Appendix A2: Description of trials – locally advanced patients, sequential CT

Data on progression-free survival was available for 37% of the patients in the trials comparing RT + Sequential CT vs. RT.

Trial	Period of recruitment	Number of patients (in surrogate analysis)	Drugs used and dose per cycle (mg/m²)	Radiotherapy dose (Gy) / fractions
CALGB 8433 [A2-1]	1984-87	180<br>(180)	Cisplatin 100 + Vinblastine 5<br>2 cycles ; neo-adjuvant	60 Gy, 2 Gy/f, 6-7 W
EORTC 08842 [A2-2]	1984-89	75<br>(73)	Cisplatin 100 + Vindesine 3<br>2 cycles ; neo-adjuvant + alternated	55 Gy, 20 f
CEBI 138 [A2-3]	1983-89	353<br>(353)	Cisplatin 100 + Cyclophosphamide 200 + Vindesine 1.5 + Lomustine 50<br>3 cycles (+ 3 cycles if no progression) ; neo-adjuvant + adjuvant	65 Gy, 26 f, 45 D
RTOG 8808 – ECOG 4588 [A2-4]	1989-92	326<br>(326)	Cisplatin 100 + Vinblastine<br>neo-adjuvant	60 Gy, 2 Gy/f, 6W
BLT adjuvant [A2-5]<br><br>BLT neoadjuvant [A2-5]	1995-01	119 adjuvant<br>(119)<br>169 neoadjuvant<br>(169)	Cisplatin 50 + Mitomycin C 6 + Ifosfamide 3<br>or Cisplatin 50 + Mitomycin C 6 + Vinblastine 6<br>or Cisplatin 80 + Vindesine 3<br>or Cisplatin 80 + Vinorelbine 30<br>3 cycles ; adjuvant or neo-adjuvant	not standardized, followed local practice
GMMA Ankara 1995 [A2-6]	1995-96	30<br>(30)	Cisplatin 40 + Etoposide 200 + Ifosfamide 200<br>2 cycles ; neo-adjuvant	36 Gy 12 f, split course 7 D free, 12.5 Gy 5 f
Taxane Only<br>Tax SI009 [A2-7]	1995-99	208<br>(208)	Docetaxel 100<br>maximum of 3 cycles ; neo-adjuvant	Local treatment (surgery or radiotherapy) decided at baseline by the clinician

BLT: Big Lung Trial. CALGB: Cancer and Leukemia Group B. CEBI: Carcinome épidermoïde bronchique inopérable. ECOG: Eastern Cooperative Oncology Group. EORTC: European Organisation for Research and Treatment of Cancer. GMMA: Gülhane Military Medicine Academy. RTOG: Radiotherapy Oncology Group. Tax: Taxotere. D: days. f: fractions. Gy: Grays. W: weeks.

1. Dillman R, Seagren S, Propert K et al. A randomised trial of induction chemotherapy plus high-dose radiation versus radiation alone in stage III non-small cell lung cancer. N Engl J Med 1990; 323:940-5.
2. Planting A, Helle P, Drings P et al. A randomized study of high-dose split course radiotherapy preceeded by high-dose chemotherapy versus high-dose radiotherapy only in locally advanced non-small cell lung cancer. Ann Oncol 1996; 7:139-44.
3. Le Chevalier T, Arriagada R, Quoix E et al. Radiotherapy alone versus combined chemotherapy and radiotherapy in nonresectable non-small cell lung cancer: First analysis of a randomized trial in 353 patients. J Natl Cancer Inst 1991; 83(6):417-23.
4. Sause W, Kolesar P, Taylor IV S et al. Final results of phase III trial in regionally advanced unresectable non-small cell lung cancer. Chest 2000; 117:358-64.
5. Fairlamb D, Milroy R, Gower N, Parmar M, Peake M, Rudd R, Souhami R, Spiro S, Stephens R, Waller D. A randomised comparison of radical radiotherapy with or without chemotherapy for patients with non-small lung cancer: results from the Big Lung Trial. Radiother Oncol. 2005 ;75:134-40.
6. Ulutin HC, Güden M, Oysul K et al. Split-course radiotherapy with or without concurrent or sequential chemotherapy in non-small cell lung cancer. Radiat Med 2000; 18:93-96.
7. Mattson K, Abratt R, ten Velde G et al. Docetaxel as neoadjuvant therapy for radically treatable stage III non-small cell lung cancer: a multinational randomised phase III study. Ann Oncol 2003;14:116-22.

Appendix A3: Description of trials – locally advanced patients, concurrent CT

Data on progression-free survival was available for 85% of the patients in the trials comparing RT + Concurrent CT vs. RT.

Trial	Period of recruitment	Number of patients (in surrogate analysis)	Drugs used and dose per cycle (mg/m²)	Radiotherapy dose (Gy) / fractions
EORTC 08844 [A3-1]	1984-89	331 (331)	Arm 2 : Cisplatin 30 – 4 cycles<br>Arm 3: Cisplatin 6 daily x 20	55 Gy, 20 f, 7 W
PMCI 88 C091 [A3-2]	1989-95	208 (208)	Arm 3: Carboplatin 70<br>Arm 4: Carboplatin 70	60 Gy, 30 f, 3 (tid) or 6 W
CALGB-ECOG [A3-3]	1991-94	282 (282)	Carboplatin 100 – 6 cycles	60 Gy, 30 f, 6 W
NKB-CKVO 9411 [A3-4]	1994-98	160 (159)	Carboplatin 840 ci during 6 weeks	60 Gy, 30 f, 6 W
NPC IIIB 96-01[A3-5]	1996-2003	584 (580)	Carboplatin 15 daily x 33<br>Induction CT in both arms	66 Gy, 33 f, 6 W
GMMA Ankara 1995 [A2-6]	1995-96	30 (30)	Cisplatin 6 daily	48.5 Gy, 17 f, sc
JCOG 9812 [A3-6]	1999-01	46 (46)	Carboplatin 30 daily x 20	60 Gy, 30 f, 6 W
Kragujevac 88 [A3-7]	1988-89	169 (169)	Carboplatin 100 + Etoposide 100 Arm II : Weekly<br>Arm III : 3 cycles	64.8 Gy, 54 f bid, 5.4 W
Kragujevac 90 [A3-8]	1990-91	131 (131)	Carboplatin 50 + Etoposide 50	69.6 Gy, 58 f bid, 5.8 W
NCCTG 90 24 51 [A3-9]	1992-93	74 (74)	Cisplatin 30 + Etoposide 100	60 Gy, 40 f bid, 4 W sc
LAMP ACR 427 [A3-10]	1998-2001	177 (174)	Carboplatin AUC2 + Paclitaxel 45 mg/m² weekly<br>Induction CT in both arms	63 Gy, 34 f, 7 W
Uludag [A3-11]	1996-2001	45 (45)	Paclitaxel 30-60	RT: 59.4 Gy, 1.8 Gy/f<br>RT-CT: 63 Gy, 1.8 Gy/f
GMMA Ankara 97 [A3-12]	1995-96	51 (51)	Paclitaxel 60	48.5 Gy, 17 f, sc
Brocat Study Group CT/RT 99/97	1997-2002	212 (212)	Paclitaxel 60	60-66 Gy, 30-33 fr
TAX 206 trial	1999-2001	89 (60)	Docetaxel 20	60 Gy, 30 fr

CALGB: Cancer and Leukemia Group B. ECOG: Eastern Cooperative Oncology Group. EORTC: European Organisation for Research and Treatment of Cancer. GMMA: Gülhane Military Medicine Academy. JCOG: Japan Clinical Oncology Group. LAMP ACR: Locally Advanced Multi-Modality Protocol American College of Radiology. NCCTG: North Central Cancer Treatment Group. NKB-CKVO: Nederlandse Kanker Bestrijding – Commissie voor Klinisch Vergelijkend Onderzoek. NPC IIIB: Non Petites Cellules stage IIIB. PMCI: Peter MacCallum Cancer Institute. TAX: Taxotere.
AUC: area under the curve. bid: two fractions per day. ci: continuous infusion. CT: chemotherapy. f: fractions. Gy: Grays. sc: split course. tid: three fractions per day. W: weeks.

1. Schaake-Koning C et al. Effects of concomitant cisplatin and radiotherapy on inoperable non small cell lung cancer. N Engl J Med 1992; 326: 524-30.
2. Ball D et al. A randomised phase III study of accelerated or standard fraction radiotherapy with or without concurrent carboplatin in inoperable non-small cell lung cancer: final report of an Australian multi-centre trial. Radiother Oncol 1999; 52: 129-36.
3. Clamon G et al. Radiosensitization with carboplatin for patients with unresectable stage III non small cell lung cancer : a phase III trial of the Cancer and Leukemia Group B and the Eastern Cooperative Oncology Group. J Clin Oncol 1999; 17:4-11.
4. Groen H et al. Continuously infused carboplatin used as radiosensitizer in locally unresectable non-small-cell lung cancer: a multicenter phase III study. Ann Oncol 2004; 15: 427-432.

5. Douillard J et al. Randomized phase III trial for stage III unresectable non small cell lung cancer: induction chemotherapy [vinorelbine-cisplatine] followed by conventional radiotion without or with daily carboplatin. Final results. Study CRG/BMS/NPC/96 of the French Lung Cancer Study Group FNCLCC and IFCT. Proceedings of the 11th World Conference on Lung Cancer. Barcelone 2005; S16:O-040.

6. Shinji Atagi et al. Standard Thoracic Radiotherapy With or Without Concurrent Daily Low-dose Carboplatin in Elderly Patients with Locally Advanced Non-small Cell Lung Cancer: a Phase III Trial of the Japan Clinical Oncology Group (JCOG9812). Jpn J Clin Oncol 2005; 35(4):195-201.

7. Jeremic B et al Randomized trial of hyperfractionated radiation therapy with or without concurrent chemotherapy for stage III non small cell lung cancer. J Clin Oncol 1995; 13:452-8.

8. Jeremic B et al. Hyperfractionated radiation therapy with or without concurrent low-dose daily carboplatin/etoposide for non small cell lung cancer: a randomized study. J Clin Oncol 1996; 14:1065-70.

9. Bonner J et al. The possible advantage of hyperfractionated thoracic radiotherapy in the treatment of locally advanced non small cell lung cancer. Results of a North Central Cancer Treatment Group phase III study. Cancer 1998; 82:1037-48.

10. Belani CP et al. Combined Chemoradiotherapy Regimens of Paclitaxel and Carboplatin for Locally Advanced Non–Small-Cell Lung Cancer: A Randomized Phase II Locally Advanced Multi-Modality Protocol. J Clin Oncol 2005; 23:5883-91.

11. Sarihan S et al. Randomized phase II study of radiotherapy alone versus radiotherapy with paclitaxel in non-small cell lung cancer. J Int Med Res 2004; 32:375-83.

12. Ulutin HC, Pak Y. Preliminary results of radiotherapy with or without weekly paclitaxel in locally advanced non-small cell lung cancer. J Cancer Res Clin Oncol 2003; 129:52-6.

Appendix A4: Description of trials – locally advanced patients, sequential versus concurrent CT

Data on progression-free survival was available for 100% of the patients in the trials comparing RT + Sequential CT vs. RT + Concurrent CT.

Trial	Period of recruitment	Number of patients (in surrogate analysis)	Comments	Randomised chemotherapy	Radiotherapy
WJLCG [A4-1]	1992-94	314 (313)	Unresectable stage III neoadjuvant vs concurrent	Cisplatin 80 + Vindesine 3 + Mitomycin C 8	56 Gy, 28 f
RTOG 9410 [A4-2]	1994-98	407 (407)	neoadjuvant vs concurrent	Cisplatin 100 + Vinblastine 5<br>5 cycles	60 Gy
GLOT-GFPC NPC 95-01 [A4-3]	1996-2000	205 (202)	IIIA N2, IIIB neoadjuvant vs concurrent+ adjuvant	Cisplatin 120 + Vinorelbine 30<br>versus<br>Cisplatin 20 + Etoposide 50<br>then Cisplatin 80+ Vinorelbine 30	66 Gy, 33 f, 6.5 W
EORTC 08972 [A4-4]	1999-2003	158 (158)	Inoperable stage I, II, III neoadjuvant vs concurrent	2 cycles : Cisplatin 75 + Gemcitabine 1250 – 2 cycles<br>versus<br>Cisplatin 6 daily	66 Gy, 24 f, 32 D
CALGB 8831 [A4-5]	1988-1989	91 (91)	Adjuvant vs concurrent	Cisplatin 100 + Vinblastine 5<br>4 cycles<br>versus<br>Carboplatin 100 – 6 cycles	60 Gy, 2 Gy/f, 6 W
GMMA Ankara 1995 [A2-6]	1995-96	30 (30)	arm 2 vs 3 neoadjuvant vs concurrent	Cisplatin 40 + Etoposide 200 + Ifosfamide 200 – 2 cycles<br>vs<br>Cisplatin 6 daily	48.5 Gy, 17 f, sc

CALGB: Cancer and Leukemia Group B. EORTC: European Organisation for Research and Treatment of Cancer. GMMA: Gülhane Military Medicine Academy. GLOT-GFPC NPC: Groupe Lyon-Saint-Etienne d'Oncologie Thoracique–Groupe Français Non Petites Cellules. RTOG: Radiotherapy Oncology Group. WJLCG: West Japan Lung Cancer Group.
D: days. f: fractions. Gy: Grays. sc: split course. W: weeks.

1. Furuse K, Fukuoka M, Kawahara M et al. Phase III study of concurrent versus sequential thoracic radiotherapy in combination with mitomycin, vindesine, and cisplatin in unresectable stage III non-small cell lung cancer. J Clin Oncol 1999; 17:2692-9.

2. Curran W, Scott C, Langer C et al. Long-term benefit is observed in a phase III comparison of sequential versus concurrent chemo-radiation for patients with unresected stage III NSCLC: RTOG 9410. Proceedings of ASCO 2003; 22:621.

3. Fournel P, Robinet G, Thomas P et al. Randomized Phase III Trial of Sequential Chemoradiotherapy Compared With Concurrent Chemoradiotherapy in Locally Advanced Non-Small-Cell Lung Cancer: Groupe Lyon-Saint-Etienne d'Oncologie Thoracique-Groupe Francais de Pneumo-Cancérologie NPC 95-01 Study. J Clin Oncol 2005; 23:5910-7.

4. Belderbos J, Uitterhoeve L, van Zandwijk N et al. on behalf of the EORTC LCG and RT Group. Randomised trial of sequential versus concurrent chemo-radiotherapy in patients with inoperable non-small cell lung cancer (EORTC 08972-22973). Eur J Cancer 2007; 43:114-21.

5. Clamon G, Herndon J, Eaton W et al. A feasibility study of extended chemotherapy for locally advanced non-small cell lung cancer: a phase II trial of Cancer and Leukemia Group B. Cancer Invest 1994; 12:273-282.

Appendix A5: Description of trials – radiotherapy trials

Data on progression-free survival was available for 100% of the patients the trials in the MAR-LC meta-analysis.

Trial	Period of recruitment	Number of patients (in surrogate analysis)	Chemotherapy	Randomised radiotherapy
ECOG 3588 [A5-1,*]	1989-1992	417<br>(417)	Cisplatin 60 mg/m² d1<br>Etoposide 120 mg/m² d1,2,3<br>4 cycles (3 w)	Standard: 45 Gy / 25 f / 5 w<br>Experimental: 45 Gy / 30 f / 3 w bid
NCCTG 892052 [A5-2,*]	1990-1996	268<br>(268)	Cisplatin 30 mg/m² d1,2,3<br>Etoposide 130 mg/m² d1,2,3<br>6 cycles‡ (4 w)	Standard: 50.4 Gy / 28 f / 5.5 w<br>Experimental: 48 Gy / 32 f / 5.5 w sc* bid
RTOG 8808-<br>ECOG 4588 [A2-4]	1989-1992	326<br>(326)	None	Standard: 60 Gy / 30 f / 6 w<br>Experimental: 69.6 Gy / 58 f / 6 w bid
PMCI 88C091 [A3-2]	1989-1995	101<br>(101)	None	Standard: 60 Gy / 30 f / 6 w<br>Experimental: 60 Gy / 30 f / 3 w bid
PMCI 88C091 CT [A3-2]	1989-1995	107<br>(107)	Carboplatin 70 mg/m² d1-5<br>+ Carboplatin 70 mg/m² d29-33 in standard arm	Standard: 60 Gy / 30 f / 6 w<br>Experimental: 60 Gy / 30 f / 3 w bid
CHART[A5-3]	1990-1995	563<br>(563)	None	Standard: 60 Gy / 30 f / 6 w<br>Experimental: 54 Gy / 36 f / 1.5 tid
NCCTG 902451 [A3-9]	1992-1993	74<br>(74)	None	Standard: 60 Gy / 30 f / 6 w<br>Experimental: 60 Gy / 40 f / 6 w sc bid
NCCTG 942452 [A5-4]	1994-1999	246<br>(246)	Cisplatin 30 mg/m² d1-3;28-30<br>Etoposide 100 mg/m² d1-3;28-30	Standard: 60 Gy / 30 f / 6 w<br>Experimental: 60 Gy / 40 f / 6 w sc bid
ECOG 2597 [A5-5]	1998-2001	119<br>(119)	Carboplatin AUC 6 d1<br>Paclitaxel 225 mg/m² d1<br>2 cycles£ (3 w)	Standard: 64 Gy / 32 f / 6.5 w<br>Experimental: 57.6 Gy / 36 f / 2.5 w tid
Gliwice 2001 [A5-6]	2001-2006	58<br>(58)	None	Standard: 72 Gy / 40 f / 8 w<br>Experimental: 72 Gy / 40 f / 5.5 w
CHARTWEL [A5-7]	1997-2005	300<br>(300)	None	Standard: 66 Gy / 33 f / 6·5 w<br>Experimental: 60 Gy / 40 f / 2·5 w tid
CHARTWEL CT [A5-7]	1997-2005	106<br>(106)	Neoadjuvant CT - depends on centres	Standard: 66 Gy / 33 f / 6·5 w<br>Experimental: 60 Gy / 40 f / 2·5 w tid

*: small-cell lung cancer trials.
CHART, Continuous Hyperfractionated Accelerated Radiation Therapy; CHARTWEL, CHART Week-End Less; ECOG, Eastern Cooperative Oncology Group; EORTC: European Organisation for Research and Treatment of Cancer. NCCTG, North Central Cancer Treatment Group; PCMI, Peter MacCallum Institute; RTOG, Radiation Therapy Oncology Group.
AUC: area under the curve. bid: two fractions per day. d: day. f: fractions. Gy: Grays. tid: three fractions per day. w: weeks. sc: split course. NSCLC: non–small-cell lung cancer.

1. Turrisi AT, Kim K, Blum R, Sause WT, Livingston RB, Komaki R, Wagner H, Aisner S, Johnson DH. Twice-daily compared with once-daily thoracic radiotherapy in limited small-cell lung cancer treated concurrently with cisplatin and etoposide. N Engl J Med 1999; 340:265-271.

2. Schild SE, Bonner JA, Shanahan TG, Brooks BJ, Marks RS, Geyer SM, Hillman SL, Farr GH, Tazelaar HD, Krook JE, Geoffroy FJ, Salim M, Arusell RM, Mailliard JA, Schaefer PL, Jett JR. Long-term results of a phase III trial comparing once-daily radiotherapy with twice-daily radiotherapy in limited-stage small-cell lung cancer. Int J Radiat Oncol Biol Phys 2004; 59:943-951.

3. 5. Saunders M, Dische S, Barrett A, Harvey A, Griffiths G, Parmar M. Continuous, hyperfractionated, accelerated radiotherapy (CHART) versus conventional radiotherapy in non-small cell lung cancer: Mature data from the randomised multicentre trial. Radiother Oncol 1999; 52:137-148.

4. Schild SE, Stella PJ, Geyer SM, Bonner JA, Marks RS, McGinnis WL, Goetz SP, Kuross SA, Mailliard JA, Kugler JW, Shaefer PL, Rett JR. Phase III trial comparing chemotherapy plus once-daily or twice-daily radiotherapy in stage III non-small-cell lung cancer. Int J Radiat Oncol Biol Phys 2002; 54:370–378.

5. Belani CP, Wang W, Johnson DH, Wagner H, Schiller J, Veeder M, Mehta M. Phase III study of the Eastern Cooperative Oncology Group (ECOG 2597): induction chemotherapy followed by either standard thoracic radiotherapy or hyperfractionated accelerated radiotherapy for patients with unresectable stage IIIA and B non-small-cell lung cancer. J Clin Oncol 2005; 23:3760-3767.

6. Zajusz A, Behrendt K, Nowicka E, Plewicki G, Gawkowska-Suwinska M, Giglok M, Smolska B. Early results of continuous accelerated radiotherapy (CAIR) for LA-NSCLC patients. Radiother Oncol 2006; 81(Suppl 1):S386. [Poster]

7. Baumann M, Herrmann T, Koch R, et al, on behalf of the CHARTWEL-bronchus group. Final results of the randomized phase III CHARTWEL-trial (ARO 97-1) comparing hyperfractionated-accelerated vs conventionally fractionated radiotherapy in non-small cell lung cancer (NSCLC). Eur J Cancer 2009; Supplements 7(3): 4.

Appendix A6: Cumulative percentage of DFS/PFS events

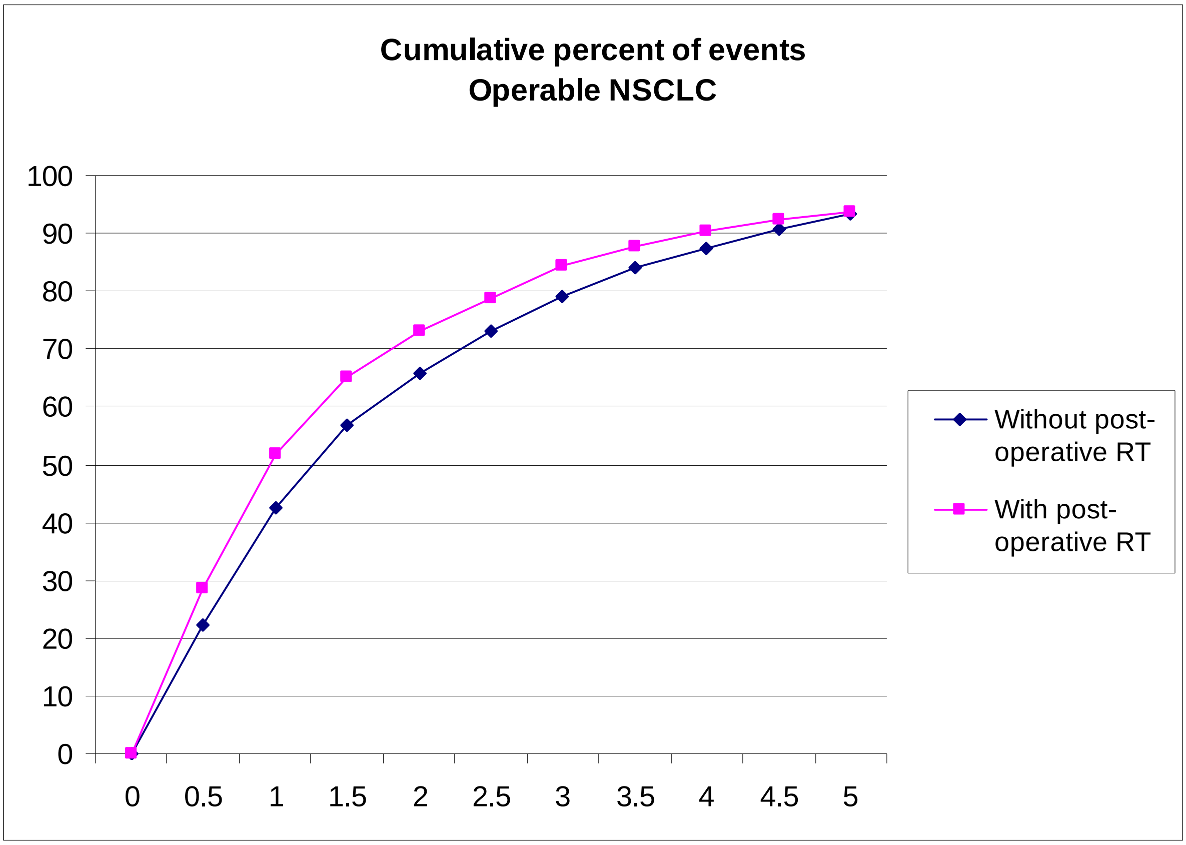

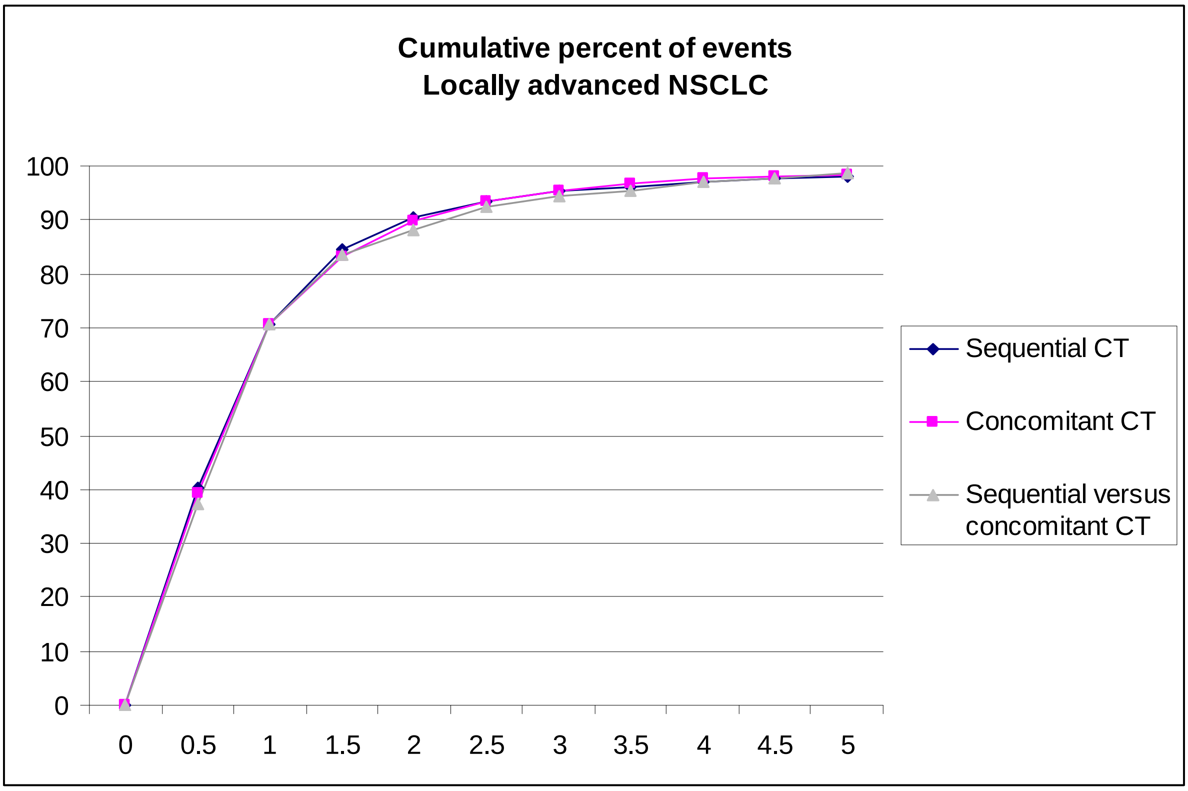

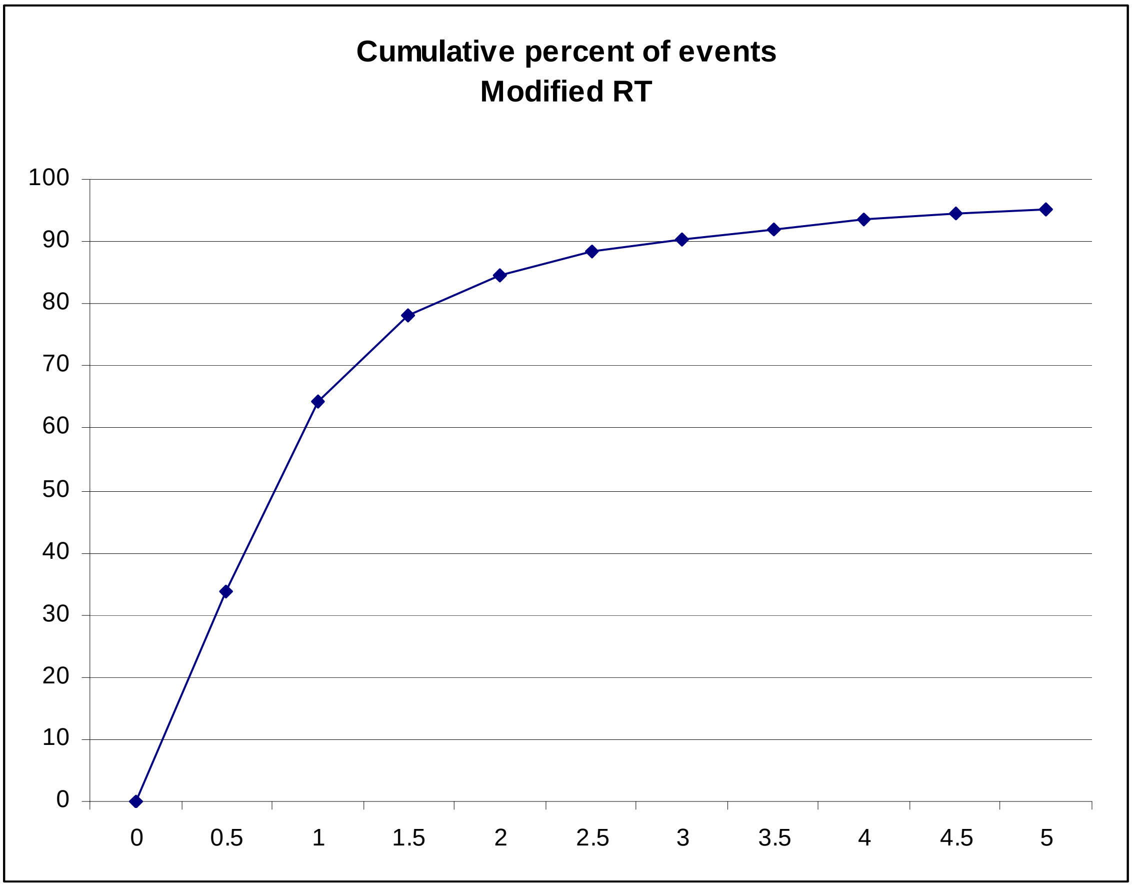
Cumulative percent of events
Modified RT
100
90
80
70
60
50
40
30
20
10
0
0
0.5
1
1.5
2
2.5
3
3.5
4
4.5
5

Appendix A7: Summary of data

Table 1: Description of analyzed data in operable NSCLC

	Patients (trials)	Any events			Deaths	
	N	N	% in first 3 years	% deaths in events* (near°)	N	% in first 5 years
Operable non-small cell lung cancer						
Without radiotherapy	5 379 (17)	2 525	79%	25% (26%)	2 163	91%
With radiotherapy	2 247 (7)	1 673	84%	29% (30%)	1 566	92%

*day of event=day of death
°near: day of death-day of event <= 7 days.

Table 2: Description of analyzed data in locally advanced lung cancer

	Patients (trials)	Locoregional events		Any events			Deaths	
	N	N	% in first 2 years	N	% in first 2 years	% deaths in events	N	% in first 5 years
Locally advanced non-small cell lung cancer								
Sequential chemotherapy	1 458 (8)	-	-	1 375	90%	30%* (33%°)	1 333	97%
Concurrent chemotherapy	2 552 (15)	744	90%	2 391	90%	25% (27%)	2 305	98%
Sequential versus concurrent chemotherapy	1 201 (6)	-	-	1 094	88%	23% (26%)	1 065	97%
Locally advanced lung cancer								
Radiotherapy	2 685 (12)	894	86%	2 562	85%	27% (33%)	2 471	94%

*day of event=day of death
°near: day of death - day of event <= 7 days.

Table 3: Follow-up and survival times

Meta-analysis	Median of follow-up (range)	Median of overall survival (range)	Median of disease-free / progression-free survival (range)	Median of duration of loco-regional control (range)
**Operable NSCLC**				
Without radiotherapy	5.7 y (0-16.7 y)	8.2 y (4 d -16.7 y)	6.4 y (0-16.7 y)	
With radiotherapy	6.4 y (1 d – 22.2 y)	2.5 y (1 d – 22.2 y)	1.6 y (1 d – 22.2 y)	
**Locally advanced NSCLC**				
Sequential chemotherapy	6.8 y (0-17.8 y)	11.7 m (3 d -17.8 y)	7.9 m (0 -17.8 y)	
Concurrent chemotherapy	5.2 y (0-15.8 y)	14.1 m (0-15.8 y)	8.1 m (0-15.8 y)	21.0 m* (3 d -15.8 y)
Sequential versus concurrent chemotherapy	6.1 y (0-11.2 y)	14.6 m (4 d – 11.2 y)	8.3 m (1 d – 11.2 y)	
Locally advanced lung cancer Radiotherapy	8.6 y (0 – 14.3 y)	15.0 m (0 – 14.3 y)	9.1 m (0 – 14.3 y)	21.3 m* (1 d – 14.3 y)

d=days; m=months; y=years;

* Median durations of loco-regional control were longer than median overall survival because all events other than loco-regional were censored.

Appendix A8: Surrogacy analyses results

Each time, two models based on Hougaard and on Clayton copulas were fitted, while the optimal model according to the Akaike's criterion was retained and presented.

Abbreviations: CI= confidence interval, DFS=disease-free survival, HR=hazard ratio, LRC=loco-regional control, OS=overall survival, PFS=progression-free survival, STE=surrogate threshold effect

Evaluating effect of adjuvant chemotherapy in operable NSCLC – without post-operative radiotherapy

Hougaard copula	Disease-free survival over the entire time range
Number of trials (number of patients)	17 (5 379)
N events OS	2 163
N events DFS	2 525
Individual level	
ρ^2	0.83 [0.83-0.83]
Trial level, unweighted	
R^2	0.92 [0.88-0.95]
Regression equation, unweighted	log(HR OS)=0.08+0.98.log(HR PFS)
STE	0.88

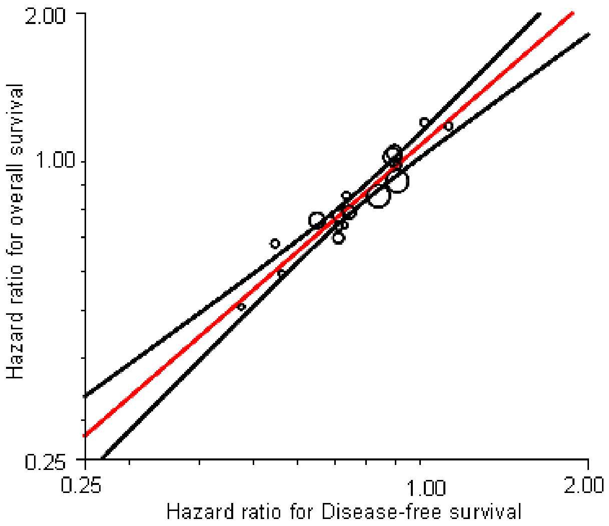

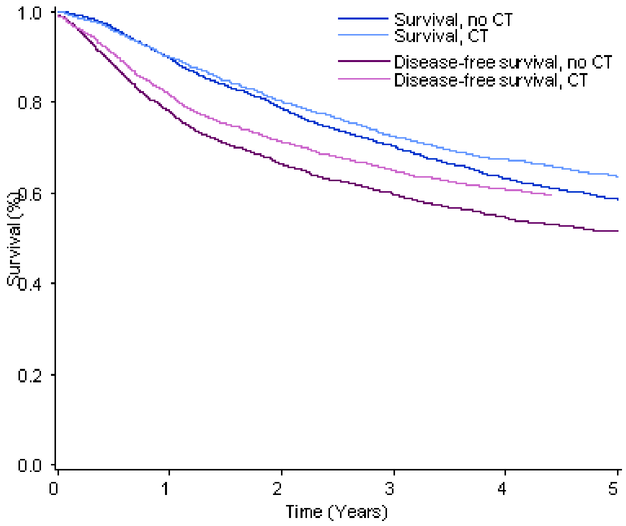

R^2 – weighted regression	Intercept [95% CI] – weighted regression	Slope [95% CI] – weighted regression
0.83	0.05 [-0.01 – 0.11]	0.91 [0.68 – 1.14]

Evaluating effect of adjuvant chemotherapy in operable NSCLC – with post-operative radiotherapy

Hougaard copula	Disease-free survival over the entire time range
Number of trials (number of patients)	7 (2 247)
N events OS	1 566
N events DFS	1 673
Individual level	
ρ^2	0.87 [0.87-0.87]
Trial level, unweighted	
R^2	0.99 [0.98-1.00]
Regression equation, unweighted	log(HR OS)=0.01+0.66.log(HR PFS)
STE	0.95

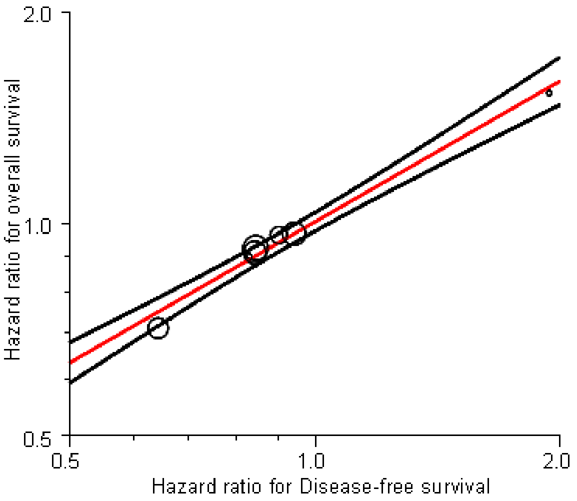

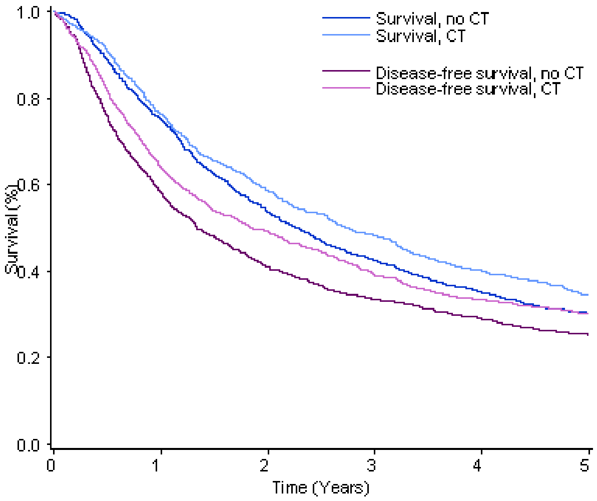

R^2 – weighted regression	Intercept [95% CI] – weighted regression	Slope [95% CI] – weighted regression
0.96	0.03 [-0.01 – 0.07]	0.77 [0.61 – 0.94]

Evaluating effect of sequential chemotherapy in locally advanced NSCLC

Clayton copula	Progression-free survival over the entire time range
Number of trials (number of patients)	8 (1 458)
N events OS	1 333
N events PFS	1 375
Individual level	
ρ^2	0.77 [0.77-0.77]
Trial level, unweighted	
R^2	0.96 [0.93-0.99]
Regression equation, unweighted	log(HR OS)=0.04+1.22.log(HR PFS)
STE	0.93

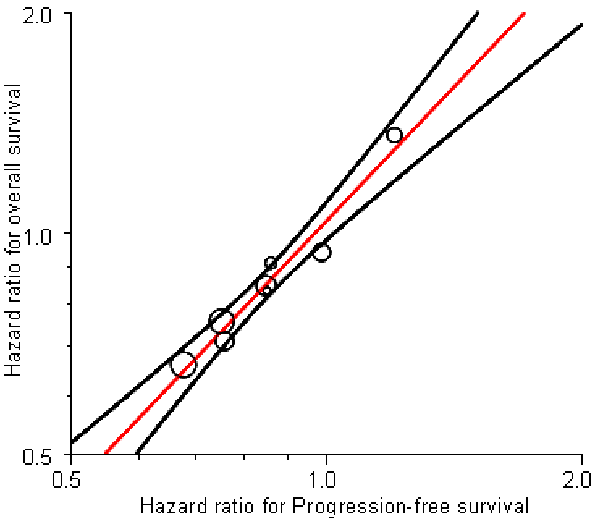

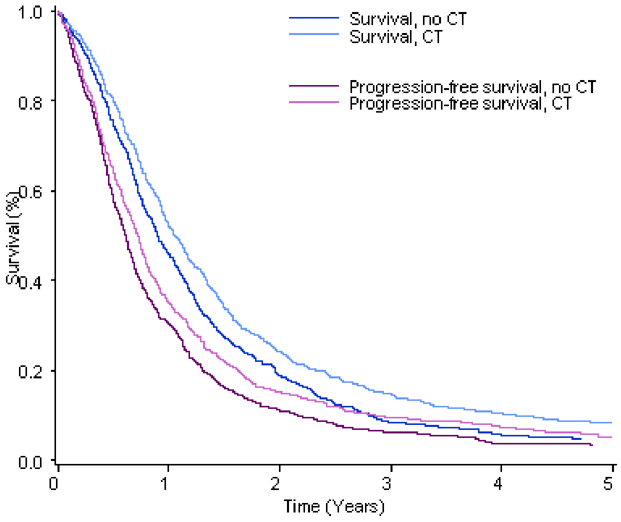

R^2 – weighted regression	Intercept [95% CI] – weighted regression	Slope [95% CI] – weighted regression
0.96	0.03 [-0.03 – 0.10]	1.16 [0.93 – 1.40]

Evaluating effect of concurrent chemotherapy in locally advanced NSCLC

Progression-free survival

Hougaard copula	Progression-free survival over the entire time range
Number of trials (number of patients)	15 (2 552)
N events OS	2 305
N events PFS	2 391
Individual level	
ρ^2	0.85 [0.85-0.85]
Trial level, unweighted	
R^2	0.97 [0.96-0.99]
Regression equation, unweighted	log(HR OS)=0.01+0.87.log(HR PFS)
STE	0.95

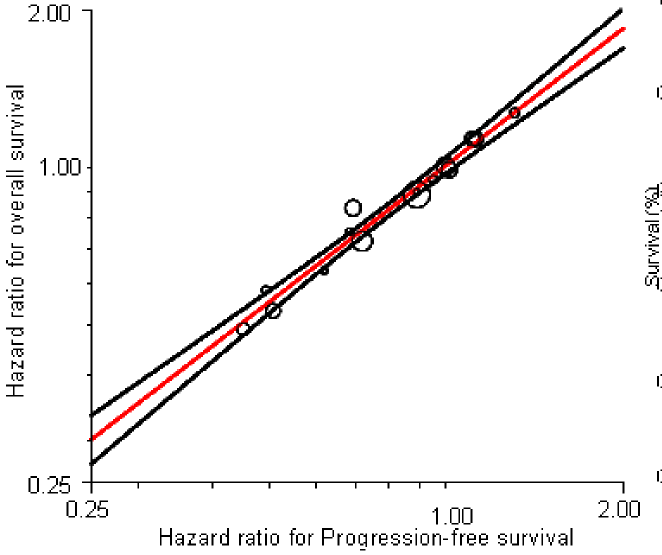

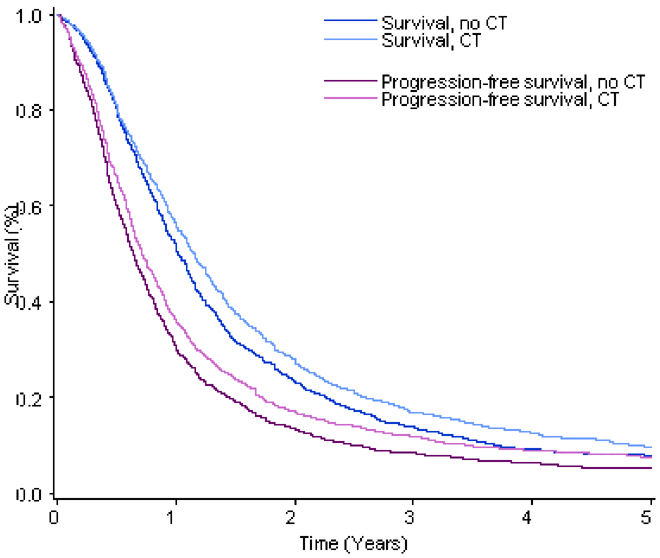

R^2 – weighted regression	Intercept [95% CI] – weighted regression	Slope [95% CI] – weighted regression
0.96	0.01 [-0.03 – 0.04]	0.88 [0.78 – 0.99]

Loco-regional control

Hougaard copula	Loco-regional control over the entire time range
Number of trials (number of patients)	13 (2 123)
N events OS	1 929
N events LRC	744
Individual level	
ρ^2	0.71 [0.71-0.71]
Trial level, unweighted	
R^2	0.85 [0.77-0.92]
Regression equation, unweighted	log(HR OS)= 0.00+0.64.log(HR LRC)
STE	0.89

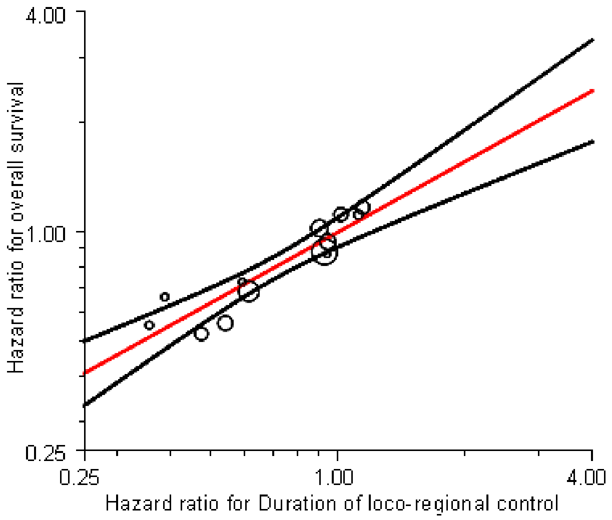

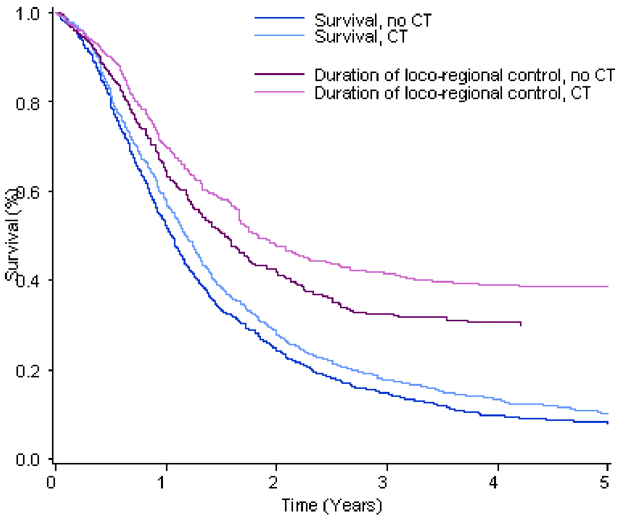

R^2 – weighted regression	Intercept [95% CI] – weighted regression	Slope [95% CI] – weighted regression
0.87	-0.01 [-0.08 – 0.07]	0.75 [0.56 – 0.94]

Evaluating effect of sequential versus concurrent chemotherapy in locally advanced NSCLC

Hougaard copula	Progression-free survival over the entire time range
Number of trials (number of patients)	6 (1 201)
N events OS	1 065
N events PFS	1 094
Individual level	
ρ^2	0.83 [0.83-0.83]
Trial level, unweighted	
R^2	0.89 [0.81-0.97]
Regression equation, unweighted	log(HR OS)=-0.01+0.85.log(HR PFS)
STE	0.90

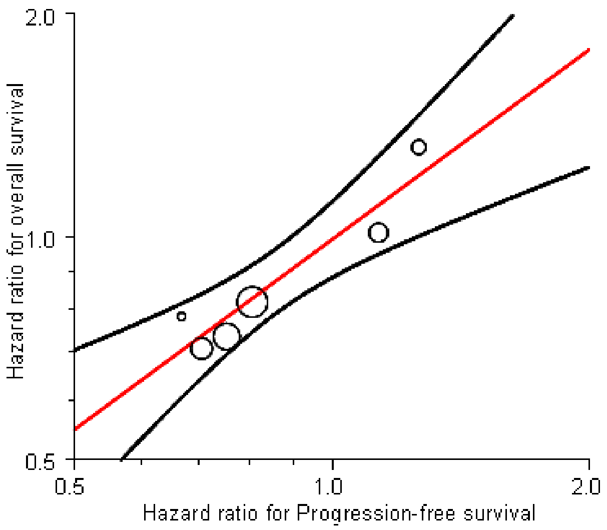

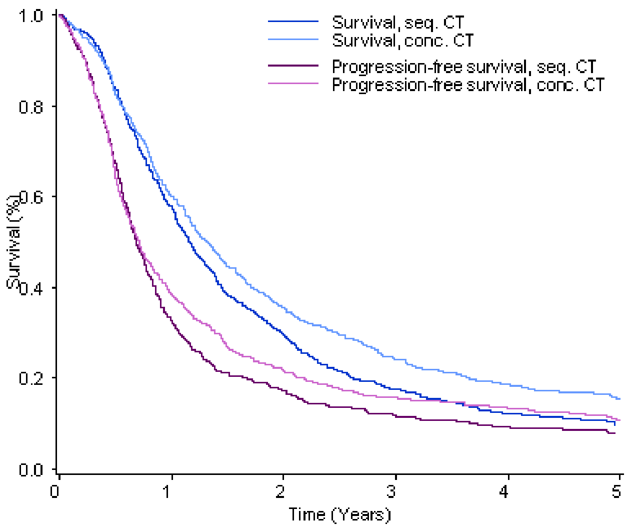

R^2 – weighted regression	Intercept [95% CI] – weighted regression	Slope [95% CI] – weighted regression
0.92	-0.03 [-0.12 – 0.06]	0.90 [0.55 – 1.25]

Evaluating effect of modified radiotherapy versus conventional radiotherapy in lung cancer

Progression-free survival

Hougaard copula	Progression-free survival over the entire time range
Number of trials (number of patients)	12 (2 685)
N events OS	2 471
N events PFS	2 562
Individual level	
ρ^2	0.81 [0.81-0.81]
Trial level, unweighted	
R^2	0.96 [0.93-0.98]
Regression equation, unweighted	log(HR OS)=-0.04+0.99.log(HR PFS)
STE	1.00

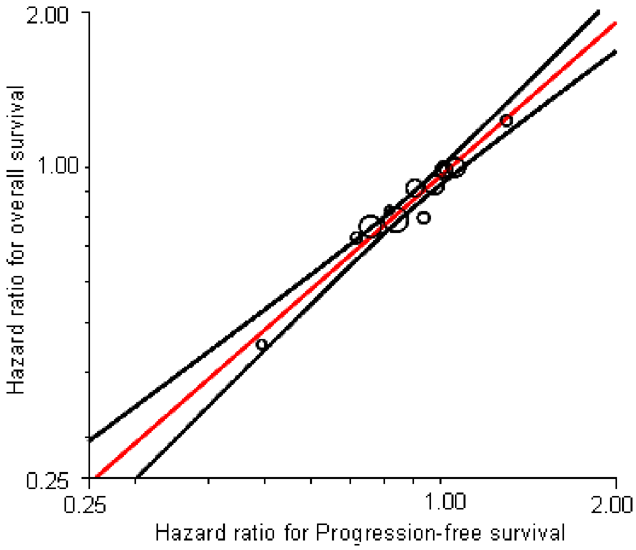

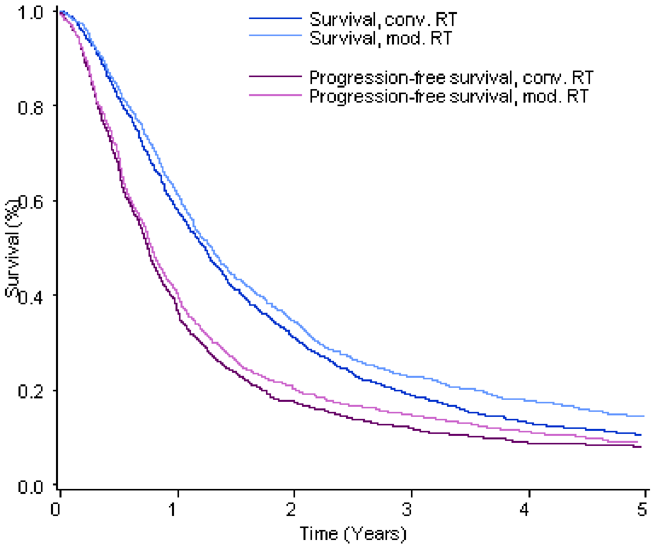

R^2 – weighted regression	Intercept [95% CI] – weighted regression	Slope [95% CI] – weighted regression
0.94	-0.04 [-0.08 – -0.01]	0.94 [0.78 – 1.11]

Loco-regional control

Hougaard copula	Loco-regional control over the entire time range
Number of trials (number of patients)	10 (2 079)
N events OS<br>N events LRC	1 915<br>894
Individual level	
ρ^2	0.61 [0.61-0.61]
Trial level, unweighted	
R^2	0.95[0.91-0.98]
Regression equation, unweighted	log(HR OS)= 0.00 +0.75.log(HR LRC)
STE	0.95

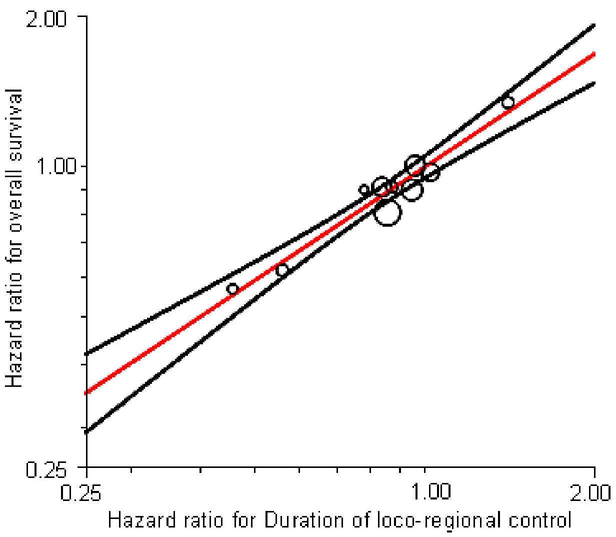

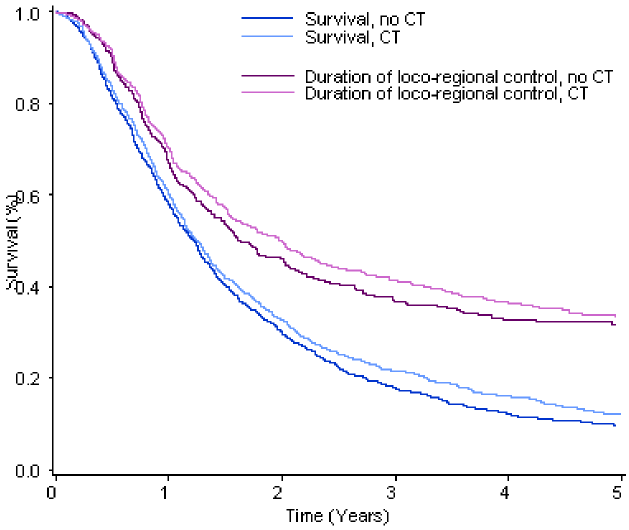

R^2 – weighted regression	Intercept [95% CI] – weighted regression	Slope [95% CI] – weighted regression
0.88	-0.03 [-0.08 – 0.03]	0.76 [0.53 – 0.99]

Summary of unweighted regression lines

Table 4: Summary of surrogacy results

	Non weighted Regression intercept	[	95%		CI	]	Non weighted Regression slope	[	95%		CI	]	STE
							**Progression/Disease-free survival**						
**Operable NSCLC**													
Without post-operative radiotherapy	0.08	[	0.02	-	0.14	]	0.98	[	0.82	-	1.14	]	0.88
With post-operative radiotherapy	0.01	[	-0.02	-	0.04	]	0.66	[	0.57	-	0.76	]	0.95
**Locally advanced NSCLC**													
Sequential chemotherapy	0.04	[	-0.02	-	0.10	]	1.22	[	0.96	-	1.48	]	0.93
Concurrent chemotherapy	0.01	[	-0.03	-	0.05	]	0.87	[	0.78	-	0.96	]	0.95
Sequential versus concurrent chemotherapy	-0.01	[	-0.13	-	0.11	]	0.85	[	0.44	-	1.27	]	0.90
**Locally advanced lung cancer**													
Radiotherapy	-0.04	[	-0.08	-	0.00	]	0.99	[	0.84	-	1.14	]	1.00
							**Loco-regional control**						
**Locally advanced NSCLC**													
Concurrent chemotherapy	0.00	[	-0.10	-	0.09	]	0.64	[	0.46	-	0.83	]	0.89
**Locally advanced lung cancer**													
Radiotherapy	0.00	[	-0.05	-	0.05	]	0.75	[	0.61	-	0.90	]	0.95

Appendix A9: Sensitivity analysis: different cut-off points

Different cut-off time points are explored for DFS/PFS. OS is censored at 5 years.

	Number of events (% of total events)	Individual level	Trial level, unweighted			STE
		ρ^2	R^2	Intercept	Slope	
**Adjuvant, CT vs no CT**						
Cut-off at 1 yr	1077 (43%)	0.66 [0.66-0.66]	0.62 [0.47-0.76]	0.02 [-0.10;0.15]	0.67 [0.38-0.96]	0.84
Cut-off at 1.5 yr	1436 (57%)	0.71 [0.71-0.71]	0.75 [0.65-0.86]	0.05 [-0.04;0.16]	0.78 [0.54-1.03]	0.85
Cut-off at 2 yr	1658 (66%)	0.74 [0.74-0.74]	0.86 [0.80-0.92]	0.07 [-0.01;0.14]	0.82 [0.64-1.00]	0.86
Cut-off at 2.5 yr	1845 (73%)	0.76 [0.76-0.76]	0.87 [0.80-0.93]	0.06 [-0.01;0.13]	0.80 [0.62-0.97]	0.86
Cut-off at 3 yr	1994 (79%)	0.77 [0.77-0.77]	0.88 [0.83-0.93]	0.06 [-0.01;0.13]	0.81 [0.65-0.97]	0.87
**Adjuvant, RT+ CT vs RT**						
Cut-off at 1 yr	869 (52%)	0.72 [0.72-0.72]	0.95 [0.92-0.99]	0.02 [-0.04;0.07]	0.61 [0.46-0.77]	0.90
Cut-off at 1.5 yr	1091 (65%)	0.76 [0.76-0.76]	0.95 [0.92-0.99]	0.01 [-0.05;0.07]	0.60 [0.45-0.76]	0.90
Cut-off at 2 yr	1221 (73%)	0.77 [0.77-0.77]	0.94 [0.90-0.98]	0.01 [-0.05;0.08]	0.59 [0.42-0.77]	0.89
Cut-off at 2.5 yr	1320 (79%)	0.79 [0.79-0.79]	0.96 [0.92-0.99]	0.02 [-0.04;0.07]	0.67 [0.50-0.84]	0.90
Cut-off at 3 yr	1411 (84%)	0.81 [0.81-0.81]	0.96 [0.93-0.99]	0.01 [-0.05;0.06]	0.66 [0.51-0.82]	0.92
**Locally advanced, RT + Seq. CT vs RT**						
Cut-off at 1 yr	969 (70%)	0.76 [0.76-0.76]	0.79 [0.67-0.92]	-0.04 [-0.14;0.07]	1.13 [0.56-1.71]	0.95
Cut-off at 1.5 yr	1160 (84%)	0.74 [0.74-0.74]	0.86 [0.77-0.95]	-0.03 [-0.10;0.05]	0.98 [0.58-1.38]	0.96
Cut-off at 2 yr	1244 (90%)	0.74 [0.74-0.74]	0.77 [0.63-0.91]	-0.02 [-0.12;0.08]	0.98 [0.44-1.52]	0.94
**Locally advanced, RT + Conc. CT vs RT**						
Cut-off at 1 yr	1689 (71%)	0.76 [0.76-0.76]	0.93 [0.90-0.97]	0.00 [-0.05;0.05]	0.75 [0.63-0.87]	0.94
Cut-off at 1.5 yr	1990 (83%)	0.77 [0.77-0.77]	0.94 [0.90-0.97]	0.00 [-0.05;0.05]	0.77 [0.65-0.89]	0.94
Cut-off at 2 yr	2149 (90%)	0.79 [0.79-0.79]	0.95 [0.92-0.97]	0.00 [-0.04;0.05]	0.80 [0.68-0.91]	0.94
**Locally advanced, RT+ Seq. CT vs RT**						
Cut-off at 1 yr	773 (71%)	0.72 [0.72-0.72]	0.64 [0.41-0.87]	-0.07 [-0.22;0.08]	0.60 [-0.02;1.22]	0.92
Cut-off at 1.5 yr	912 (83%)	0.76 [0.76-0.76]	0.70 [0.49-0.87]	-0.06 [-0.21;0.08]	0.61 [0.05-1.17]	0.91
Cut-off at 2 yr	964 (88%)	0.77 [0.77-0.77]	0.75 [0.58-0.92]	-0.05 [-0.19;0.08]	0.64 [0.13-1.16]	0.91
MAR-LC						
Cut-off at 1 yr	1644 (64%)	0.72 [0.72-0.72]	0.77 [0.66-0.88]	-0.09 [-0.16;-0.02]	0.86 [0.53-1.19]	1.02
Cut-off at 1.5 yr	2004 (78%)	0.74 [0.74-0.74]	0.83 [0.74-0.91]	-0.06 [-0.13;0.01]	1.05 [0.71-1.38]	1.00
Cut-off at 2 yr	2165 (85%)	0.76 [0.76-0.76]	0.85 [0.78-0.93]	-0.05 [-0.11;0.02]	1.02 [0.72-1.32]	0.99

CT=chemotherapy; conc=concurrent;RT=radiotherapy, sequ=sequential See appendix 8 for other abbreviations

Appendix A10: Leave-one out crossvalidation results

Operable NSCLC without post-operative radiotherapy – DFS

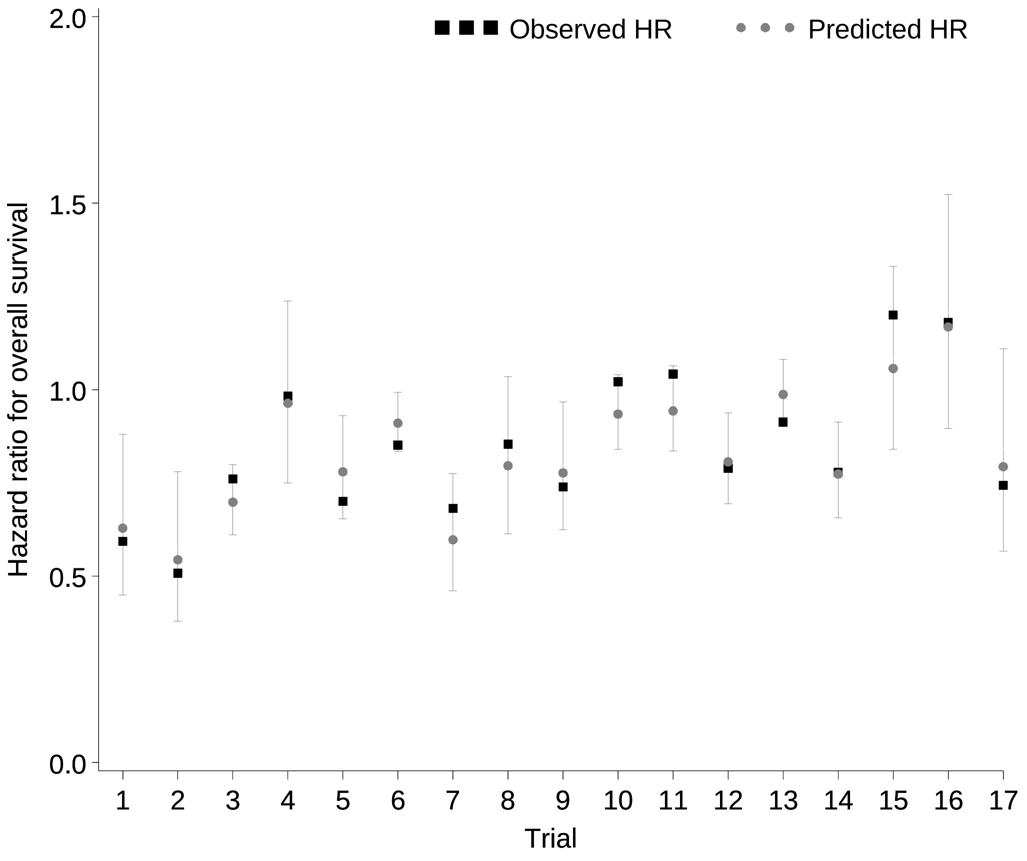

Operable NSCLC with post-operative radiotherapy - DFS

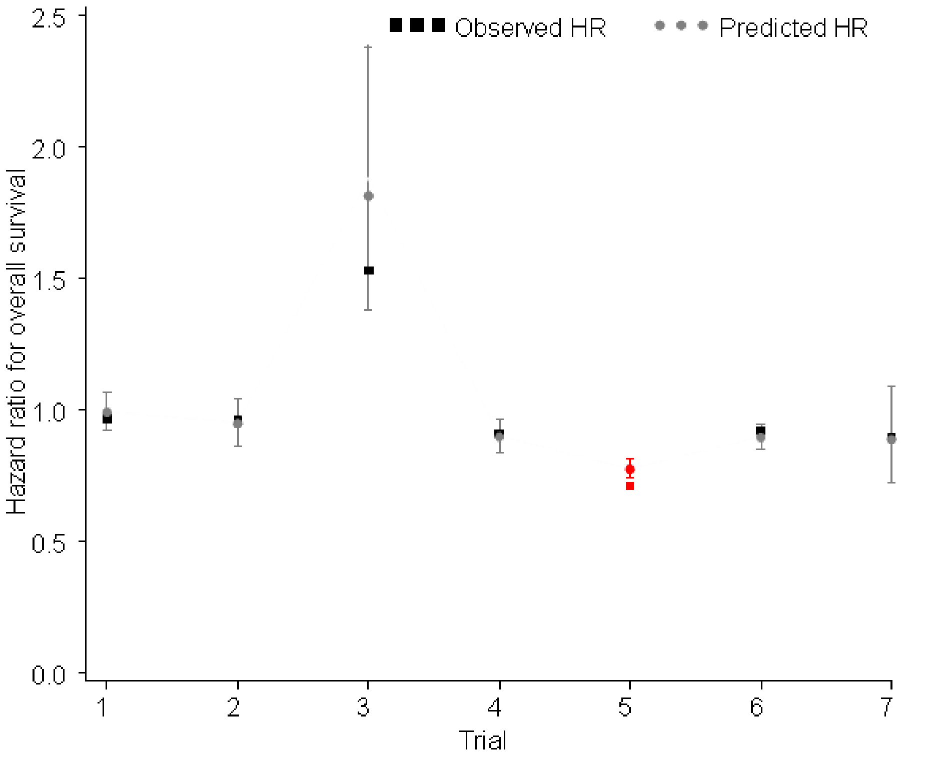

Locally advanced NSCLC, sequential chemotherapy – PFS

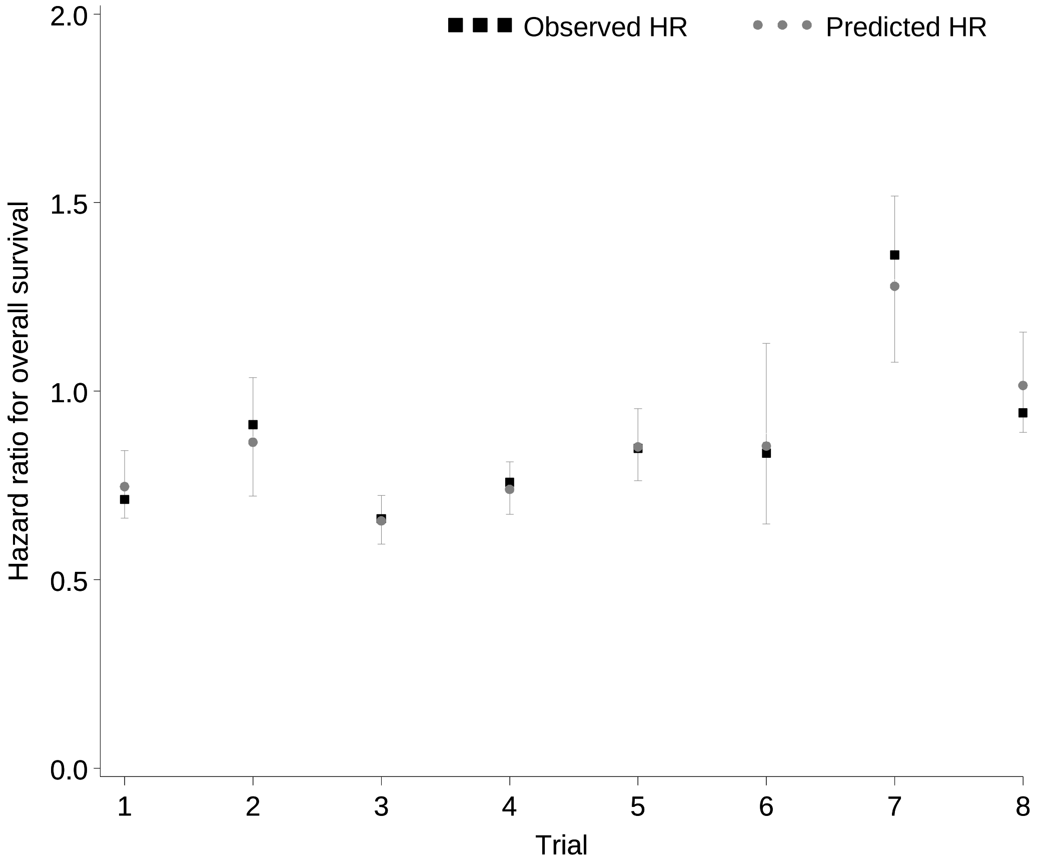

Locally advanced NSCLC, concurrent chemotherapy – PFS

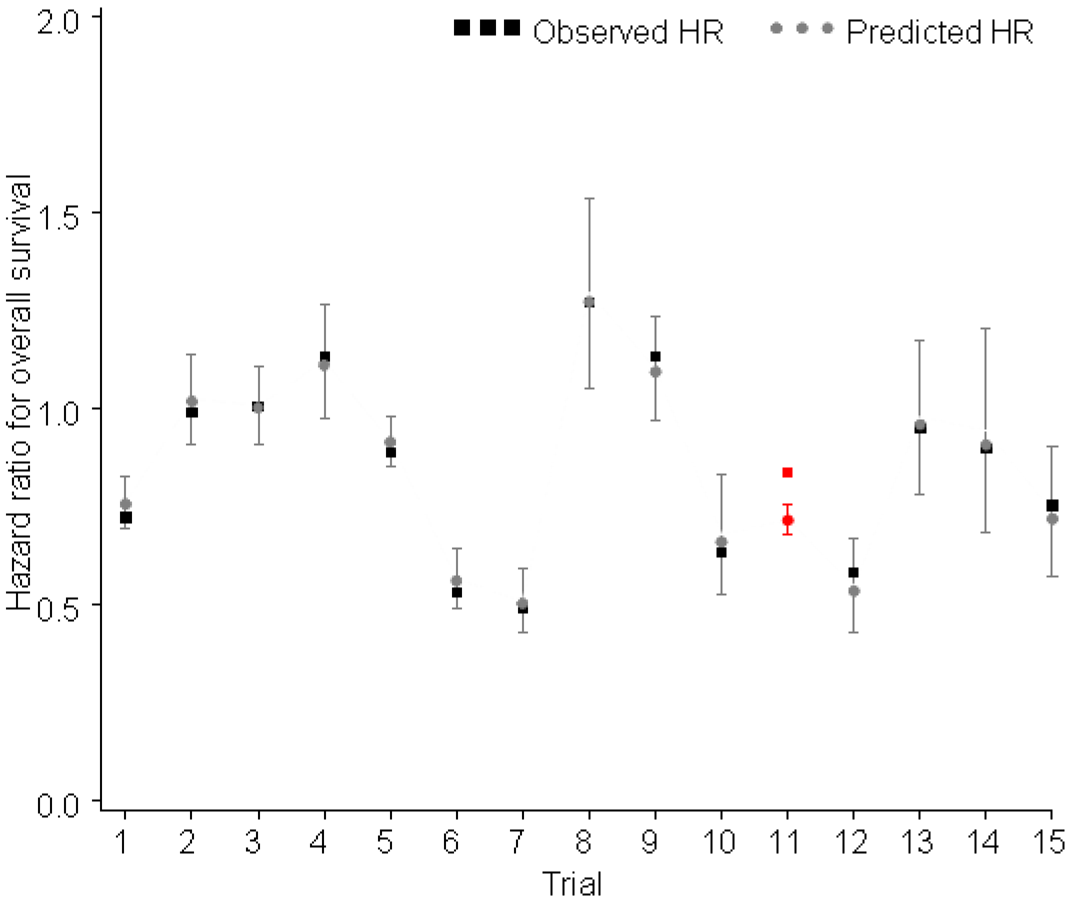

Locally advanced NSCLC, concurrent chemotherapy – LRC

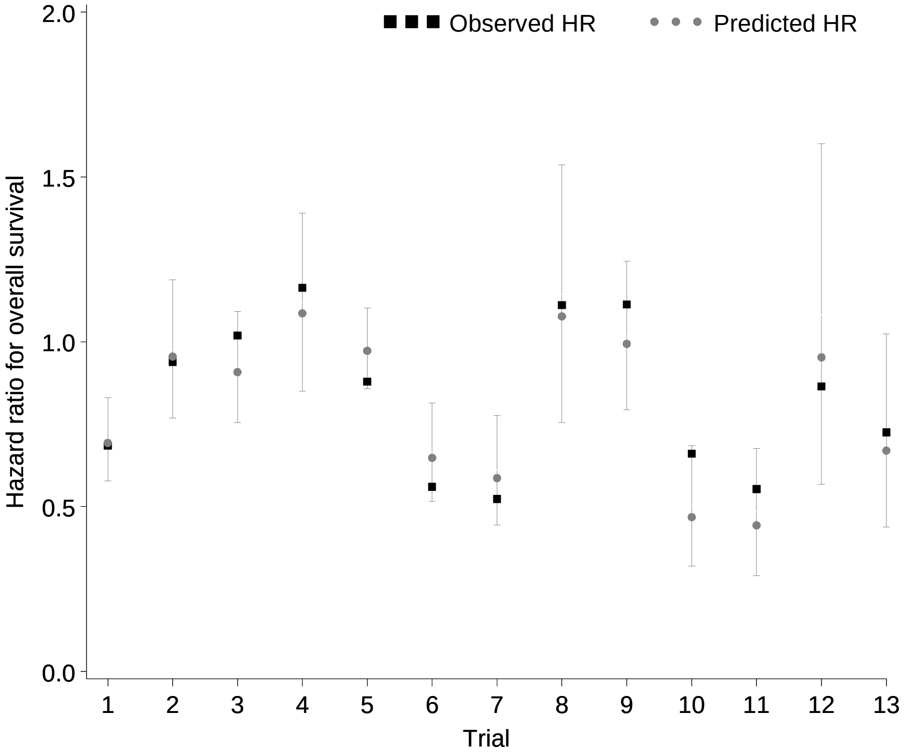

Locally advanced NSCLC, sequential versus concurrent chemotherapy – PFS

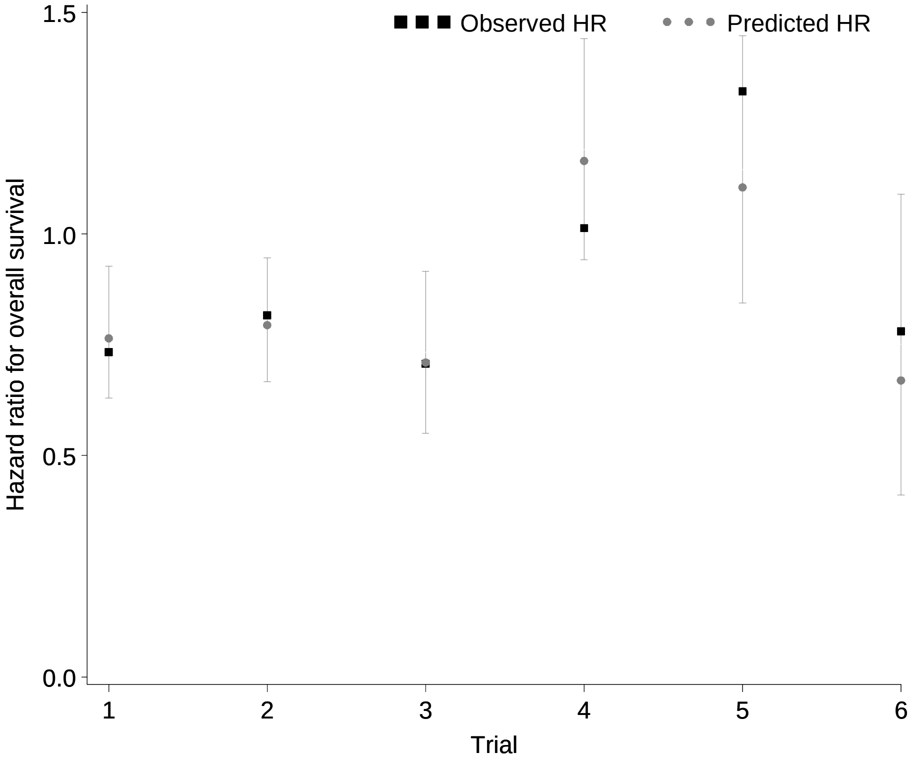

Modified radiotherapy – PFS

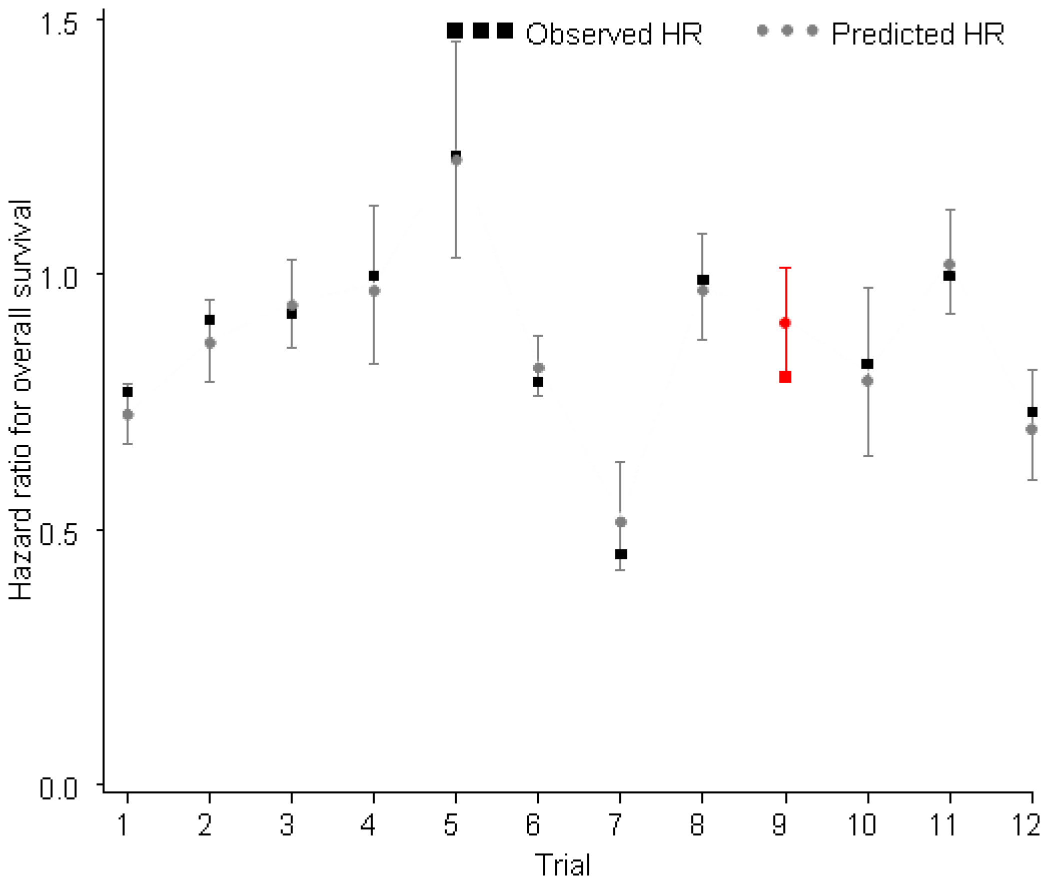

Modified radiotherapy - LRC

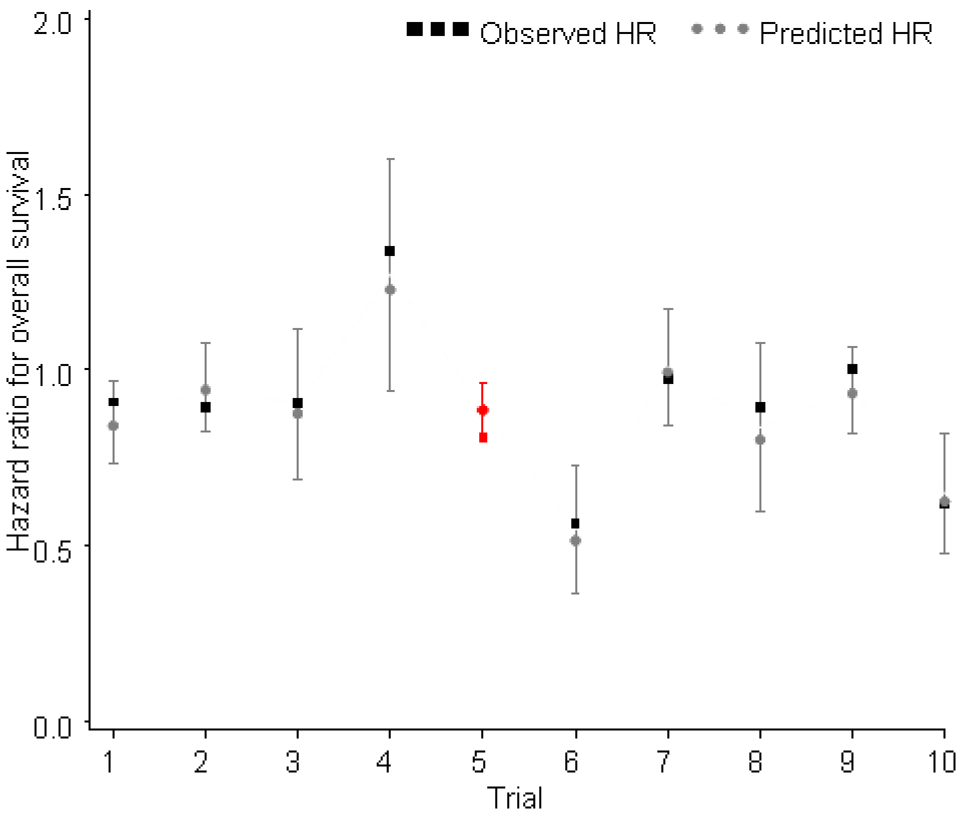

Results are for validation of DFS/PFS if no otherwise specified.

Table 5: Summary of cross-validation results

**Meta-analysis**	**Number of trials with predicted effect on OS included in the 95% prediction interval / Total number of trials**
**Operable NSCLC**	
Without radiotherapy	17 / 17
With radiotherapy	6 / 7
**Locally advanced NSCLC**	
Sequential chemotherapy	8 / 8
Concurrent chemotherapy	14 / 15<br>(13 / 13 for LRC)
Sequential versus concurrent chemotherapy	6 / 6
**Locally advanced lung cancer**	
Radiotherapy	11 / 12<br>(9/10 for LRC)

DFS=disease-free survival, LRC=loco-regional control, OS=overall survival, PFS=progression-free survival
